# Convergent validity and reliability of a novel repeated agility protocol in junior rugby league players

**DOI:** 10.12688/f1000research.23129.1

**Published:** 2020-06-17

**Authors:** Anthony Nicholls, Anthony Leicht, Jonathan Connor, Aaron Halliday, Kenji Doma

**Affiliations:** 1 Sport & Exercise Science, James Cook University, Douglas, Queensland, 4814, Australia; 2 Physical Education, Kirwan State Highschool, Kirwan, Queensland, 4817, Australia

**Keywords:** Change of direction, anaerobic power, sprint, speed, recovery rate

## Abstract

**Background: **Rugby league involves repeated, complex, change-of-direction movements, although there are no test protocols that specifically assesses these physical fitness profiles. Thus, the current study examined the convergent validity and reliability of a repeated Illinois Agility (RIA) protocol in adolescent Rugby League players.

**Methods:** Twenty-two junior Rugby League players completed 4 sessions with each separated by 7 days. Initially, physical fitness characteristics at baseline (i.e., multi-stage fitness, countermovement jump, 30-m sprint, single-effort agility and repeated sprint ability [RSA]) were assessed. The second session involved a familiarisation of RIA and repeated T-agility test (RTT) protocols. During the third and fourth sessions, participants completed the RIA and RTT protocols in a randomised, counterbalanced design to examine the validity and test-retest reliability of these protocols.

**Results:** For convergent validity, significant correlations were identified between RIA and RTT performances (r= >0.80; p<0.05). For contributors to RIA performance, significant correlations were identified between all baseline fitness characteristics and RIA (r = >0.71; p < 0.05). Reliability of the RIA protocol was near perfect with excellent intra-class correlation coefficient (0.87-0.97), good ratio limits of agreement (×/÷ 1.05-1.06) and low coefficient of variations (1.77-1.97%).

**Conclusions:** The current study has demonstrated the RIA to be a simple, valid and reliable field test that can provide coaches with information about their athlete’s ability to sustain high intensity, multi-directional running efforts.

## Introduction

Rugby League (RL) is an intermittent, invasion type game that requires players to complete repetitive bursts of sprinting and multi-directional movements in response to the dynamic constraints of the game ^1^, typically referred to as ‘agility’ movements ^2^. Traditionally, the physical component of agility has been assessed using change-of-direction routes with shorter time of completion considered a strong determinant of agility performance ^3^. Some commonly used agility tests have included the Agility T-Test and the Illinois Agility test with both employed in many intermittent sports ^4^. However, these two assessment protocols employ a single bout approach for the agility performance measure ^4, 5^. Athletes in RL encounter repeated bursts of change-of-direction movements to defend or evade defenders during a game ^6^. Consequently, performance of repeated agility activities with brief periods of rest may be an important performance component necessary for RL athletes.

As a monitoring tool, the reliability of repeated agility protocols have been explored in a variety of sports ^7– 9^. Results from a study examining a Repeated T-Test (RTT) agility protocol in soccer players significantly correlated with anaerobic measures of power, speed and repeat-sprint ability (RSA), with excellent test-retest reliability ^9^. While a good indicator of agility, the Agility T-Test consists of a linear sprint, lateral shuffles and a backwards run, which are movements that are sporadic in RL ^9^. In fact, RL players change direction frequently and utilise evading movements ^5^ that are not replicated by the Agility T-Test. Therefore, the Illinois Agility test may be more reflective of the evading activities undertaken in RL, as the protocol includes vigorous changes in direction by weaving in and out of cones ^4^. As the validity and reliability of the Repeated Illinois Agility (RIA) test have yet to be determined, reporting these properties would be essential for widespread usability ^3^.

The aims of this study were three-fold: 1) to examine the convergent validity of a novel RIA test with the repeated Agility T-test protocol (i.e. RTT); 2) to identify contributors of RIA performance by comparing its measures to speed, anaerobic capacity and recovery dynamics (i.e. RSA); and 3) to determine the test-retest reliability of the RIA protocol. It was hypothesised that the RIA would demonstrate acceptable convergent validity and reliability as a repeated agility test, with relationships identified between results of the RIA and the RTT, speed, and anaerobic capacity protocols. Identification of the convergent validity and reliability of the RIA will provide coaches with a tool to assist in monitoring and training RL athletes as well as in talent development and identification.

## Methods

### Research design

**Figure 1.**
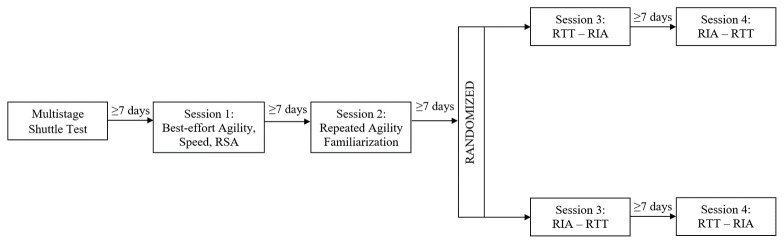
Schematic of the research design consisting of four sessions, including best-effort agility, speed, repeat sprint ability (RSA), repeated T-test agility (RTT) and repeated Illinois agility (RIA) measures.

The current study was a randomised, counter-balanced study conducted across five sessions from June, 2018 to August, 2018 ( Figure 1). During the first session, the participants completed a Multistage Shuttle test to determine predicted maximal aerobic capacity (VO _2max_) ^10^. The second session was utilised to obtain baseline assessments of speed (30-metre sprint), agility (Illinois Agility test, Agility T-Test) and repeat-sprint ability (RSA). The third session familiarised participants with the RTT and RIA tests. During the fourth and fifth sessions, participants undertook both the RIA and RTT, in randomised order, with at least 15-minutes of recovery between each protocol.

At the start of each session, muscle soreness rating was collected prior to performing a standardised warm up, using a 1-10 visual analogue scale, with 1 and 10 indicating ‘no soreness’ and ‘very, very sore’ ^11^. Participants then performed a progressive warm-up consisting of jogging for 3–5 minutes and 15-metre sprints at 50%, 70% and 100% of maximal effort. A countermovement jump (CMJ) test (Yard Stick, Swift Performance, Queensland, Australia) was then conducted to assess leg power ^12^, which was also repeated before the second agility test to confirm recovery between the repeated agility tests.

### Participants

In total, 22 adolescent, male, RL players (age 16.2 ± 0.8 yrs; body mass 80.7 ± 16.3 kg; height 1.77 ± 0.7 m) who engaged in the School of Athletic Excellence program were recruited via word of mouth, flyers and liaison with sporting teams. The participants were injury-free with at least 2 years of RL experience. According to an *a priori* calculation ^13^, a sample size of 22 was sufficient to identify significant differences in repeated-agility performance (power of 80%, alpha level of 0.05). Participants were instructed to avoid strenuous physical activity and caffeine for up to 12 hours before each testing session. All protocols were approved by the Institutional Human Research Ethics Committee and written informed consent was received from the participants and their parents/guardians prior to partaking this study (Approval number H7248).

### Multistage shuttle test

For the multistage shuttle test, participants ran back and forth in time with a series of audio signals on a 20m indoor court ^10^. The time between audio signals progressively decreased during the test resulting in an increased effort and running speed for athletes each minute. Predicted VO _2max_ was estimated using a previously developed regression equation ^10^.

### Countermovement jump test

The countermovement jump protocol was measured with a vertical jump apparatus, based on 1 cm increments (Yard Stick, Swift Performance, Queensland, Australia). To ensure standardisation of the countermovement jump test, participants were instructed to draw their arms backwards upon the eccentric phase, then swing the arms forward during the concentric phase to gain momentum and maximise the stretch-shortening cycle mechanics ^14^. The participants attempted three countermovement jumps, with approximately 30–60 seconds of rest in-between, and the highest jump reported.

### 30-m Sprint and Agility protocols

**Figure 2.**
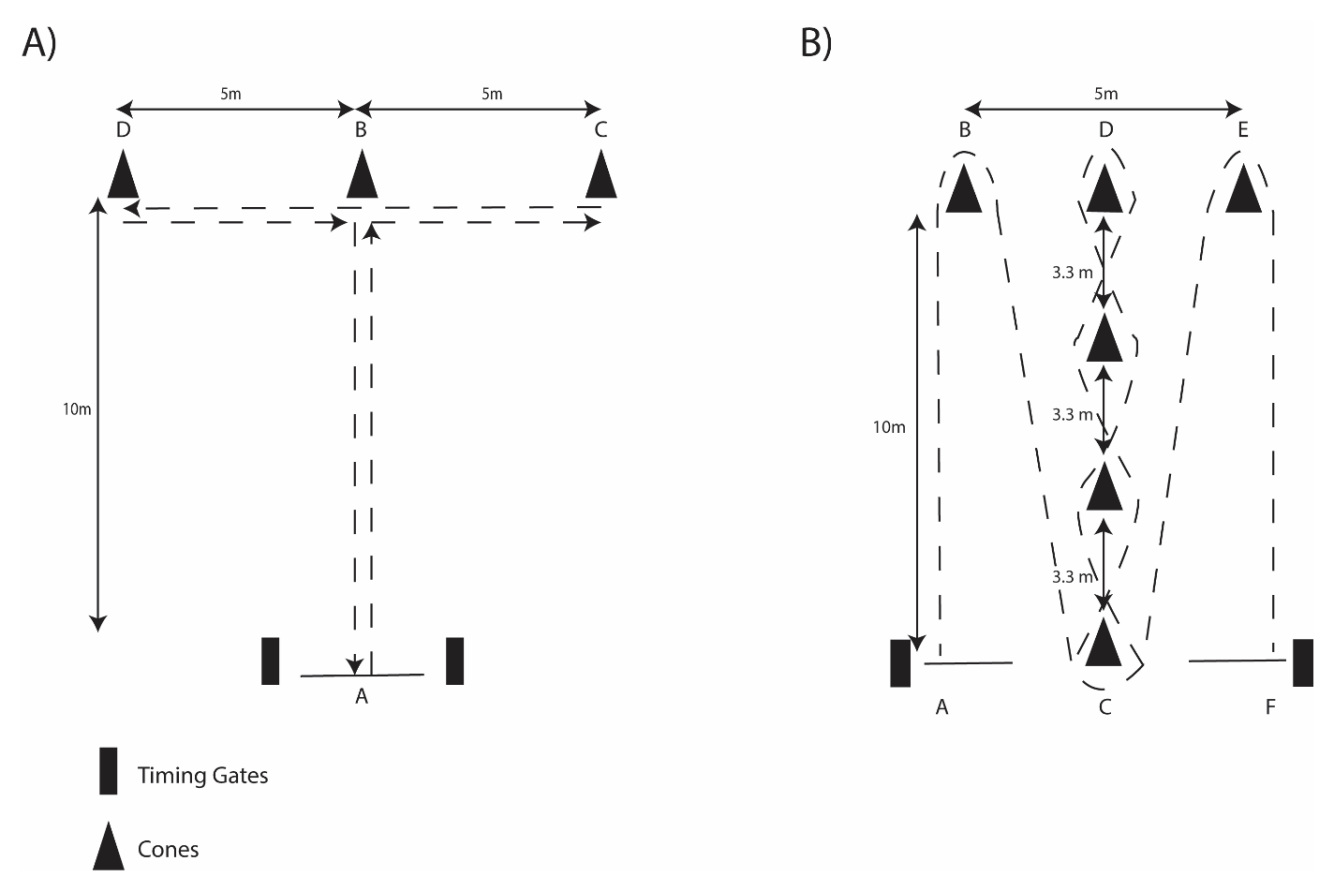
Schematic of **A**) T-Test Agility and **B**) Illinois Agility protocols.

Assessment of speed was achieved by completing 30-m maximal sprints, the Agility T-test protocol was set up within a 10-m × 10-m figure-T course ( Figure 2A), and the Illinois Agility protocol consisted of a 10m × 5m course ( Figure 2B) ^3^. To ensure protocol familiarity, the participants completed three trials at sub-maximal effort followed by one final maximal trial, with each trial interspersed by two minutes of recovery. Trial completion times were recorded using an electronic timing gate system (Speedlight Timing Gates, Swift Performance, Australia) positioned at the start/finishing line. The fastest time was used for later analysis.

### Repeat Sprint and Agility Protocols

The RSA, RTT and RIA protocols were completed by repeating the previously described protocols (i.e. 30-m sprint, T-test and Illinois Agility, respectively) across 6 cycles with varying recovery periods in-between each cycle. Specifically, each cycle within the RSA, RTT and RIA was separated by 20-, 35- and 60-second recovery, respectively, with work-to-rest ratios of approximately 1:3 ^8, 9^. Immediately after each repeated agility cycle, participant’s heart rate (HR, Polar Heart Rate Monitor, Polar H10, Finland) and maximum rating of perceived-exertion (RPE, Borg category scale 1–10) were then averaged across the 6 cycles for analysis ^15^. The following parameters were also calculated for each repeated agility protocol: total time (TT) of 6 cycles, best cycle time (BT), the average cycle time (AT) and fatigue index (FI) ^8^. FI was calculated as follows ^9^:

$$Fatigue\;Index=\left(\left(\frac{TT}{BT\times 6}\right)\times 100\right)-100$$

### Statistical analysis

Data was analysed using a statistical software (IBM SPSS version 25, Chicago, Illinois) and reported as mean ± standard deviation. Normality of the data was assessed using the Kolmogorov-Smirnov statistic. Convergent validity of the repeated agility protocols was identified via Pearson’s product moment correlation coefficients for RTT and RIA measures (i.e., TT, BT, AT and FI) and construct validity with aerobic capacity, leg power, speed and agility variables (i.e., VO _2max_, CMJ, 30-m sprint time, T-Test and Illinois Agility, respectively). The cut-off for acceptable convergent validity was established when the association was statistically significant with an r-value of ≥ 0.50 ^16^. Reliability of the repeated agility measures was determined via a paired T-test, intraclass correlation coefficients (ICC, SPSS 2-way mixed, 95% confidence intervals), coefficient of variation (CV, 95% confidence intervals) and systematic bias/ratio with 95% limits of agreement (LOA) ^17^. Where significant relationships existed between the mean difference and average of test-retest values (i.e. heteroscedastic errors), variables were transformed (natural logarithm) prior to the calculation of measurement bias/ratio × / ÷ ratio LOA ^18^. The level of significance for all analyses was set at 0.05. Finally, effect size (Cohen’s *d*) with 95% CI was used to calculate the magnitude of differences in muscle soreness and CMJ measures between RIA and RTT protocols to determine whether the recovery periods were appropriate. The ES classifications were set as small, moderate and large with values of 0.2, 0.5 and 0.8, respectively (Cohen, 1988).

## Results

For convergent validity, significant correlations were identified between RIA and most RTT variables ( Table 1 ^19^). For contributors to RIA performance, significant correlations were identified with RSA, 30-m sprint time, best effort agility measures, aerobic capacity and CMJ ( Table 2 ^19^).

**Table 1.** Relationship between performance measures of the repeated Illinois agility test (RIA), repeated T-agility test (RTT) and repeated sprint ability (RSA).

	RIA				RSA			
	TT (s)	BT (s)	AT (s)	FI (%)	TT (s)	BT (s)	AT (s)	FI (%)
RTT								
TT (s)	0.84 ***	0.81 ***	0.84 ***	0.51 ***	0.70 **	0.51 *	0.70 **	0.55 **
BT (s)	0.81 ***	0.81 ***	0.81 ***	0.44 ***	0.71 **	0.54 *	0.71 **	0.50 *
AT (s)	0.84 ***	0.80 ***	0.84 ***	0.51 ***	0.70 **	0.51 *	0.70 **	0.55 **
FI (%)	0.43 **	0.32	0.43 **	0.48 **	0.28	0.13	0.28	0.37
RSA								
TT (s)	0.80 ***	0.73 ***	0.80 ***	0.55 ***	–	–	–	–
BT (s)	0.63 ***	0.51 ***	0.63 ***	0.56 ***	–	–	–	–
AT (s)	0.80 ***	0.73 ***	0.80 ***	0.55 ***	–	–	–	–
FI (%)	0.48 **	0.61 ***	0.49 **	0.28	–	–	–	–

TT = total time; BT = best time; AT = average time; FI = fatigue index *P<0.05 **P<0.01 ***P<0.001

**Table 2.** Pearson correlation coefficients between repeated performances, perceptual and physiological indices (repeated Illinois agility [RIA], repeated T-agility test [RTT]) with aerobic capacity, leg power, speed, and agility test performance measures.

	VO _2max_ (mL·kg ^-1^·min ^-1^)	CMJ (cm)	Sprint 30m (sec)	IA (sec)	TTA (sec)
RIA					
TT (s)	-0.73 **	-0.85 **	0.89 **	0.87 **	0.72 **
BT (s)	-0.71 **	-0.79 **	0.81 **	0.86 **	0.71 **
AT (s)	-0.73 **	-0.85 **	0.89 **	0.87 **	0.72 **
FI (%)	-0.43 *	-0.57 **	0.61 **	0.49 *	0.40
HR _Avg_	-0.43	-0.09	0.34	0.24	0.38
HR _Max_	-0.20	-0.03	0.12	0.04	0.21
RPE _Avg_	0.17	0.17	-0.21	-0.16	-0.21
RPE _Max_	-0.04	-0.23	0.27	0.35	0.21
RTT					
TT (s)	-0.68 **	-0.76 **	0.80 **	0.84 **	0.80 **
BT (s)	-0.65 **	-0.74 **	0.80 **	0.86 **	0.85 **
AT (s)	-0.68 **	-0.76 **	0.80 **	0.84 **	0.80 **
FI (%)	-0.41	-0.37	0.34	0.28	0.14
HR _Avg_	-0.41	-0.05	0.26	0.13	0.23
HR _Max_	-0.33	-0.68	0.21	0.06	0.24
RPE _Avg_	0.00	0.02	-0.03	0.02	-0.07
RPE _Max_	0.02	0.04	-0.03	0.03	-0.03

CMJ = countermovement jump; TT= total time; BT = best time; AT = average time; FI = fatigue index ; Sprint 30m = 30 metre sprint; IA = Illinois Agility test; TTA = T-test agility; VO _2max_ = maximal aerobic capacity , RPE _Avg_= Average Rate of Perceived Exertion, RPE _Max_ = Maximum Rate of Perceived Exertion, HR _Avg_= Heart rate average, HR _Max_ = Maximum heart rate *P<0.05 **P<0.01 ***P<0.001

Muscle soreness ratings between the third (2.0 ± 1.5) and fourth (2.6 ± 1.7) were not significantly different (p = 0.10), with a small ES (0.37). Jump height prior to each repeated agility protocol remained unchanged between the first and second CMJ tests in the third (43.8cm ± 8.7cm and 45.4cm ± 8.4cm, respectively, p = 0.09) and fourth (44.1cm ± 9.3cm and 44.0cm ± 8.6cm, respectively, p=0.80) session, also with small ES (0.19 and 0.01, respectively).

All RIA measures were similar between sessions except for FI and maximum RPE ( Table 3 ^19^). Most RIA performance measures exhibited excellent test-retest reliability (ICC = 0.92–0.97), good levels of agreement (ratio LOA = 1.05–1.06) and low measurement error (CV = 2.17–2.68%, Table 3 ^19^). However, FI and average RPE demonstrated moderate test-retest reliability (ICC = 0.87 and 0.76, respectively), poorer levels of agreement (ratio LOA = 2.57 and 2.23, respectively) and higher measurement error (CV = 25.3 and 15.8%, respectively, Table 3 ^19^).

**Table 3.** Test-retest results, intra-class correlation coefficients (ICC, 95% confidence interval (CI)), measurement bias/ratio (log-transformed data) (×/÷ 95% ratio limits of agreement (ratio-LOA)) and within-subject coefficient of variation (95 % CI) of the repeated Illinois Agility (RIA) and T-test (RTT) protocol.

	Test (s)	Retest (s)	p	ICC (95% CI)	CV% (95% CI)	Bias ratio-LOA
RIA						
TT (s)	108.22 ± 9.14	107.38 ± 8.39	0.23	0.97 (0.92 - 0.99) ***	1.97 (0.91-2.16)	1.01 ×/ 1.06
BT (s)	17.00 ± 1.03	17.05 ± 1.05	0.60	0.96 (0.90 - 0.98) ***	1.77 (0.98-1.83)	1.00 ×/ 1.05
AT (s)	18.04 ± 1.52	17.90 ± 1.40	0.23	0.97 (0.92 - 0.99) ***	1.97 (0.91-2.16)	1.01 ×/ 1.06
FI (%)	6.02 ± 3.50	4.91 ± 3.18	0.03 †	0.87 (0.68 - 0.95) ***	25.3 (22.9-40.1)	1.32 ×/ 2.57
RPE _Avg_	4.9 ± 1.2	4.3 ± 1.7	0.07	0.76 (0.41-0.90) **	15.8 (6.1-25.6)	1.20 ×/ 2.23
RPE _Max_	6.5 ± 1.6	6.1 ± 1.7	0.04 †	0.93 (0.83-0.97) ***	8.1 (4.8-11.7)	1.08 ×/ 1.34
HR _Avg_ (bpm)	183.8 ± 8.5	180.2 ± 10.2	0.09	0.92 (0.89-0.97) ***	2.10 (1.48-2.72)	1.02 ×/ 1.06
HR _Max_ (bpm)	189.0 ± 8.3	188.3 ± 9.6	0.53	0.94 (0.83-0.98) ***	1.31 (0.78-1.84)	1.00 ×/ 1.05
RTT						
TT (s)	68.69 ± 4.79	69.01 ± 5.15	0.61	0.91 (0.79 - 0.96) ***	2.68 (1.91-1.39)	1.00 ×/ 1.08
BT (s)	11.01 ± 0.7	11.06 ± 0.74	0.58	0.91 (0.78 - 0.96) ***	2.17 (1.55-2.80)	1.00 ×/ 1.08
AT (s)	11.45 ± 0.80	11.50 ± 0.86	0.61	0.91 (0.79 - 0.96) ***	2.68 (1.91-3.14)	1.00 ×/ 1.08
FI (%)	3.98 ± 1.68	3.97 ± 1.89	0.99	0.69 (0.25 - 0.87) **	27.6 (15.3-33.7)	1.03 ×/ 2.59
RPE _Avg_	3.2 ± 1.2	3.6 ± 1.4	0.10	0.89 (0.73-0.95) ***	15.3 (8.4-22.2)	0.91 ×/ 1.85
RPE _Max_	4.4 ± 1.7	4.9 ± 1.8	0.02 †	0.93 (0.84-0.97) ***	12.3 (4.7-19.8)	0.90 ×/ 1.78
HR _Avg_ (bpm)	176.2 ± 7.5	174.0 ± 13.0	0.35	0.79 (0.45-0.92) **	2.88 (1.70-4.04)	1.02 ×/ 1.11
HR _Max_ (bpm)	186.3 ± 8.0	183.7 ± 10.0	0.20	0.68 (0.14-0.88) *	2.38 (1.06-3.69)	1.02 ×/ 1.10

TT= total time; BT = best time; AT = average time; FI = fatigue index, RPE _Avg_ = Average Rate of Perceived Exertion, RPE _Max_ = Maximum Rate of Perceived Exertion, HR _Avg_ = Heart rate average, HR _Max_ = Maximum heart rate P<0.05 **P<0.01 *** P<0.001

† Significantly different (p<0.05)

For the RTT, excellent test-retest reliability (ICC = 0.91), good levels of agreement (ratio LOA = 1.08) and low measurement error (CV = 2.17–2.68%) were identified for a few variables ( Table 3 ^19^). However, high test-retest reliability (ICC = 0.93), lower levels of agreement (ratio LOA = 1.78) and higher levels of measurement error (CV = 12.3%) were observed for maximum RPE ( Table 3 ^19^). In addition, FI, average RPE and maximum HR displayed moderate to large reliability (ICC = 0.69 – 0.89), poorer agreement (ratio LOA = 1.10 – 2.59) and higher measurement error (CV = 2.38 – 27.6%) compared to the RIA protocol ( Table 3 ^19^).

## Discussion

The current findings demonstrated strong correlations between the RIA and RTT protocols, specifically the BT, TT and AT measures. These results highlight that most time-derived measures (i.e., BT, TT and AT measures) of the RIA are replicable to a previously established repeated agility protocol, but with movement demands more representative of RL. In addition, the TT and BT of the RIA was strongly associated with the TT and BT of the RSA, indicating that the ability to maintain linear speed would result in superior performances in the RIA protocol, possibly due to similar metabolic demands ^7^. Comparable findings were reported by Fessi, Makni ^9^, with strong correlations identified between the BT and TT of their repeated agility protocol and RSA protocols in 45 team-sport athletes. Collectively, our results and others ^7, 9^, suggest that performance of repeated agility relies heavily upon the anaerobic system, a metabolic pathway predominant in RL ^20^.

The current study also identified strong test-retest reliability for time-derived measures (i.e., BT, TT and AT) of the RIA, with minimal measurement error. However, the measurement error was substantially higher for FI, confirming previous studies that reported substantially stronger reliability measures for BT, TT and AT compared to that of FI from various repeated agility protocols ^7, 8, 21^. It has been suggested that FI may exhibit weaker reproducibility as the measure is multifactorial and dependent on the stability of other variables (i.e., TT and BT) ^7, 22^. Subsequently, we, and others ^7, 8, 21, 22^, recommend that time-derived measures be primarily evaluated during repeated agility protocols.

Another novelty of the current study was the reliability of the psychophysiological responses during both RIA and RTT protocols. The test-retest reliability values for HR and RPE ranged between questionable-to-excellent classifications according to ICC scores for both RIA and RTT. However, distinctly greater measurement error and bias was observed for RPE when compared to HR measures for both RIA and RTT. These findings were similar to previous studies with poorer reliability for RPE than HR measures during various running protocols ^23– 25^. It has been postulated that HR has better stability across days given that it is an objective measure, compared to the highly subjective RPE ^26^. It has also been reported that participant’s prior knowledge of the number of sprints during repeated sprint-type protocols may affect results due to pacing ^27^. Accordingly, HR measures may be a better physiological indicator for monitoring exercise-induced stress during repeated agility protocols.

An additional, yet essential finding of this study was the relationship between baseline characteristics and performances measures from the repeated agility tests. Measures of CMJ, best-effort speed and best-effort agility correlated significantly with the time-derived variables of the RIA. These relationships indicated that lower limb power, linear speed and change-of-direction capabilities were contributing factors to successful repeated agility performances and key attributes needed for RL athletes ^28^. Our findings aligned with those of Haj-Sassi, Dardouri ^8^, who reported strong correlations between measures of jump performance and repeated agility performance with an Agility T-test protocol. These authors suggested that larger jumping performances reflected athlete’s superior ability to generate force into the ground and therefore a significantly greater change-of-direction ability ^8^. The significance of this finding attests to lower limb power production being a critical component of repeated agility performance, especially within the RIA.

Finally, the current study identified significant correlations between VO _2max_ and RIA performance measures. These findings are similar to previous studies using various repeated agility protocols ^21, 22^ as well as RSA protocols ^29– 31^. Measures of VO _2max_ has been considered essential for repeated-sprint type protocols, due to muscular reoxygenation rate ^8, 32^, optimal capacity to remove and buffer hydrogen ions within working muscles ^33^ and efficiently replenish phosphagen stores ^34^. The findings of the present study suggest that aerobic capacity is a strong contributor to superior repeated agility efforts, further highlighting the need to optimise recovery capacities between high-intensity bouts for RL athletes.

In conclusion, the RIA protocol exhibited moderate-to-excellent test-retest reliability and low measurement error for the majority of time-derived measures and psychophysiological measures, and questionable reliability for FI. Further, this study has clearly demonstrated that repeated agility performances rely upon contributions from both anaerobic and aerobic systems with the RIA, demonstrating that the qualities required for optimal RIA performance may be representative of the physical demands in RL. The RIA protocol may provide practitioners with a simple, yet effective monitoring tool to quantify athlete’s ability to generate and sustain multi-directional efforts, and their ability to recover during intermittent activities.

## Data availability

### Underlying data

James Cook University Research Data: Convergent validity and reliability of a novel repeated agility protocol in junior rugby league players. https://doi.org/10.25903/5eb0f568fad20 ^19^

This project contains the following underlying data:

- Raw_data_De-identified.xlsx (Agility protocol data in excel format)
- Raw_data_De-identified.ods (Agility protocol data in ods format)

Data are available under the terms of the Creative Commons Attribution 4.0 International license (CC-BY 4.0).
