# Convergent validity and reliability of a novel repeated agility protocol in junior rugby league players

**DOI:** 10.12688/f1000research.23129.3

**Published:** 2021-11-22

**Authors:** Anthony Nicholls, Anthony Leicht, Jonathan Connor, Aaron Halliday, Kenji Doma

**Affiliations:** 1 Sport & Exercise Science, James Cook University, Douglas, Queensland, 4814, Australia; 2 Physical Education, Kirwan State Highschool, Kirwan, Queensland, 4817, Australia

**Keywords:** Change of direction, anaerobic power, sprint, speed, recovery rate

## Abstract

**Background: : **Rugby league involves repeated, complex, and high intensity change-of-direction (COD) movements with no existing test protocols that specifically assesses these multiple physical fitness components simultaneously. Thus, the current study examined the convergent validity of a repeated Illinois Agility (RIA) protocol with the repeated T-agility protocol, and the repeatability of the RIA protocol in adolescent Rugby League players. Furthermore, aerobic capacity and anaerobic and COD performance were assessed to determine whether these physical qualities were important contributors to the RIA protocol.

**Methods:** Twenty-two junior Rugby League players completed 4 sessions with each separated by 7 days. Initially, physical fitness characteristics at baseline (i.e., Multi-stage Shuttle test, countermovement jump, 30-m sprint, single-effort COD and repeated sprint ability [RSA]) were assessed. The second session involved a familiarisation of RIA and repeated T-agility test (RTT) protocols. During the third and fourth sessions, participants completed the RIA and RTT protocols in a randomised, counterbalanced design to examine the validity and test-retest reliability of these protocols.

**Results:** For convergent validity, significant correlations were identified between RIA and RTT performances (r= >0.80; p<0.05). For contributors to RIA performance, significant correlations were identified between all baseline fitness characteristics and RIA (r = >0.71; p < 0.05). Reliability of the RIA protocol was near perfect with excellent intra-class correlation coefficient (0.87-0.97), good ratio limits of agreement (×/÷ 1.05-1.06) and low coefficient of variations (1.8-2.0%).

**Conclusions:** The current study has demonstrated the RIA to be a simple, valid and reliable field test for RL athletes that can provide coaches with information about their team’s ability to sustain high intensity, multi-directional running efforts.

## Introduction

Rugby League (RL) is an intermittent, invasion type game that requires players to complete repetitive bursts of sprinting and change-of-direction (COD) movements in response to the dynamic constraints of the game ^1^. Traditionally, the physical component of COD has been assessed using protocols with a single bout approach for the COD performance measure considered a strong determinant of match-performance in team sports ^2– 5^. However, team sports, such as RL, encounter repeated bursts of COD movements to defend or evade defenders during a game ^6^. Consequently, performance of repeated-COD activities with brief periods of rest may be an important performance component necessary for RL athletes.

As a monitoring tool, the reliability of repeated-COD protocols have been explored in a variety of sports ^7– 9^. Results from a study examining a Repeated T-Test (RTT) agility protocol in soccer players significantly correlated with anaerobic measures of power, speed and repeat-sprint ability (RSA), with excellent test-retest reliability ^9^. While a good indicator of COD performance, the Agility T-Test consists of a linear sprint, lateral shuffles and a backwards run, which are movements that are sporadic in RL ^9^. In fact, RL players change direction frequently and utilise evading movements ^10^ that are not replicated by the Agility T-Test. Therefore, the Illinois Agility test may be more reflective of the evading activities undertaken in RL, as the protocol includes vigorous changes in direction by weaving in and out of cones ^11^. Furthermore, the majority of studies examining the reliability of repeated-COD protocols have been conducted in adult athletes, despite a previous study reporting lower reliability in younger athletes ^12, 13^. Thus, research is warranted examining the reliability of repeated COD-protocols in adolescent athletes.

In addition to reliability, there has been limited investigations into validating repeated-COD protocols. Indeed, the RTT protocol was significantly correlated with anaerobic measures of power, speed and repeat-sprint ability (RSA) ^9^, suggesting that these physical qualities were pertinent for repeated-COD performance. However, separate repeated-COD protocols have yet to be compared, which is essential as each COD protocol exhibits distinct movement demands that may be suitable for specific sports. To date, no studies have examined the validity and reliability of a repeated Illinois Agility (RIA) protocol. Reporting these properties would be essential for widespread usability in RL ^4^.

The aims of this study were three-fold: 1) to examine the convergent validity of a novel RIA test with the repeated Agility T-test protocol (i.e. RTT); 2) to identify contributors of RIA performance by correlating its measures to speed, anaerobic capacity and aerobic capacity; and 3) to determine the test-retest reliability of the RIA protocol. It was hypothesised that the RIA would demonstrate acceptable convergent validity and reliability as a repeated-COD test, with relationships identified between results of the RIA and the RTT, aerobic capacity, speed, and anaerobic capacity protocols. Examining the convergent validity of the RIA protocol will determine whether this novel assessment exhibits similar attributes to a standardised COD protocol (i.e., RTT). In addition, the reliability of the RIA will determine whether this test can be reliably adopted in practice by accounting for the inherent error of the test across repeated measurements. The quality of these psychometric properties will provide coaches with a tool to assist in monitoring and training RL athletes as well as in talent development and identification.

## Methods

### Research design

**Figure 1.**
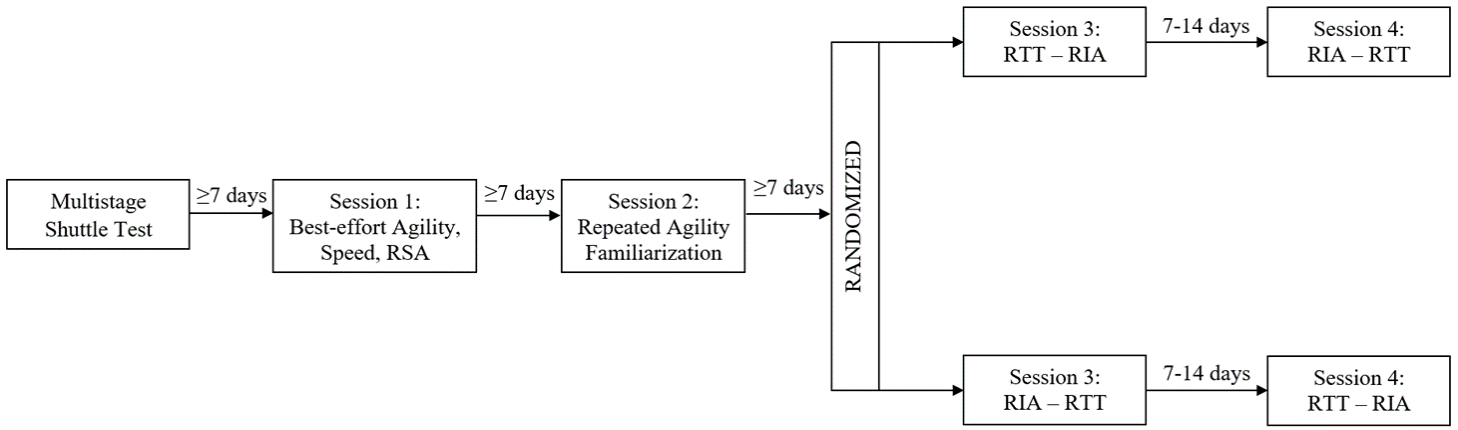
Schematic of the research design consisting of four sessions, including best-effort agility, speed, repeat sprint ability (RSA), repeated T-test agility (RTT) and repeated Illinois agility (RIA) measures.

The current study was a randomised, counter-balanced study conducted across five sessions from June, 2018 to August, 2018 ( Figure 1). During the first session, the participants completed a Multistage Shuttle test to determine predicted maximal aerobic capacity (VO _2max_) ^14^. The second session was utilised to obtain baseline assessments of speed (30-metre sprint), COD (Illinois Agility test, Agility T-Test) and repeat-sprint ability (RSA). The third session familiarised participants with the RTT and RIA tests. During the fourth and fifth sessions, participants undertook both the RIA and RTT, in randomised order, with at least 15-minutes of recovery between each protocol.

At the start of each session, muscle soreness rating was collected prior to performing a standardised warm up, using a 1–10 visual analogue scale, with 1 and 10 indicating ‘no soreness’ and ‘very, very sore’, respectively ^15^. Participants then performed a standardised warm-up consisting of jogging for 3–5 minutes and 15-metre sprints at 50%, 70% and 100% of maximal effort. A countermovement jump (CMJ) test (Yard Stick, Swift Performance, Queensland, Australia) was then conducted to assess leg power ^16^, which was also repeated before the second COD test to confirm recovery between the repeated-COD tests.

### Participants

In total, 22 adolescent, male, RL players (age 16.2 ± 0.8 yrs; body mass 80.7 ± 16.3 kg; height 1.77 ± 0.7 m) were recruited via word of mouth, flyers and liaison with sporting teams. The participants were part of the School of Athletic Excellence program, which selects and prepares students to compete at state and national competitions. The participants were injury-free with at least 2 years of RL experience. According to an *a priori* calculation ^17^, a sample size of 22 was sufficient to identify significant differences in repeated-COD performance (power of 80%, alpha level of 0.05). Participants were instructed to avoid strenuous physical activity and caffeine for up to 12 hours before each testing session. All protocols were approved by the Institutional Human Research Ethics Committee and written informed consent was received from the participants and their parent/guardian prior to partaking in this study (Approval number H7248).

### Multistage shuttle test

For the Multistage Shuttle test, participants ran back and forth in time with a series of audio signals on a 20-m indoor court in time with a series of audio signals ^14^. The time between audio signals progressively decreased during the test resulting in an increased effort and running speed for athletes each minute. Predicted VO _2max_ was estimated based on the level completed, using a previously developed regression equation ^14^.

### Countermovement jump test

The countermovement jump protocol was measured with a vertical jump apparatus, based on 1-cm increments, with the units of measure reported in cm (Yard Stick, Swift Performance, Queensland, Australia). To ensure standardisation of the countermovement jump test, participants were instructed to draw their arms backwards upon the eccentric phase, then swing the arms forward during the concentric phase to gain momentum and maximise the stretch-shortening cycle mechanics ^18^. The participants attempted three countermovement jumps, with approximately 30–60 seconds of rest in-between, and the highest jump reported.

### 30-m Sprint and Change-of-direction protocols

**Figure 2.**
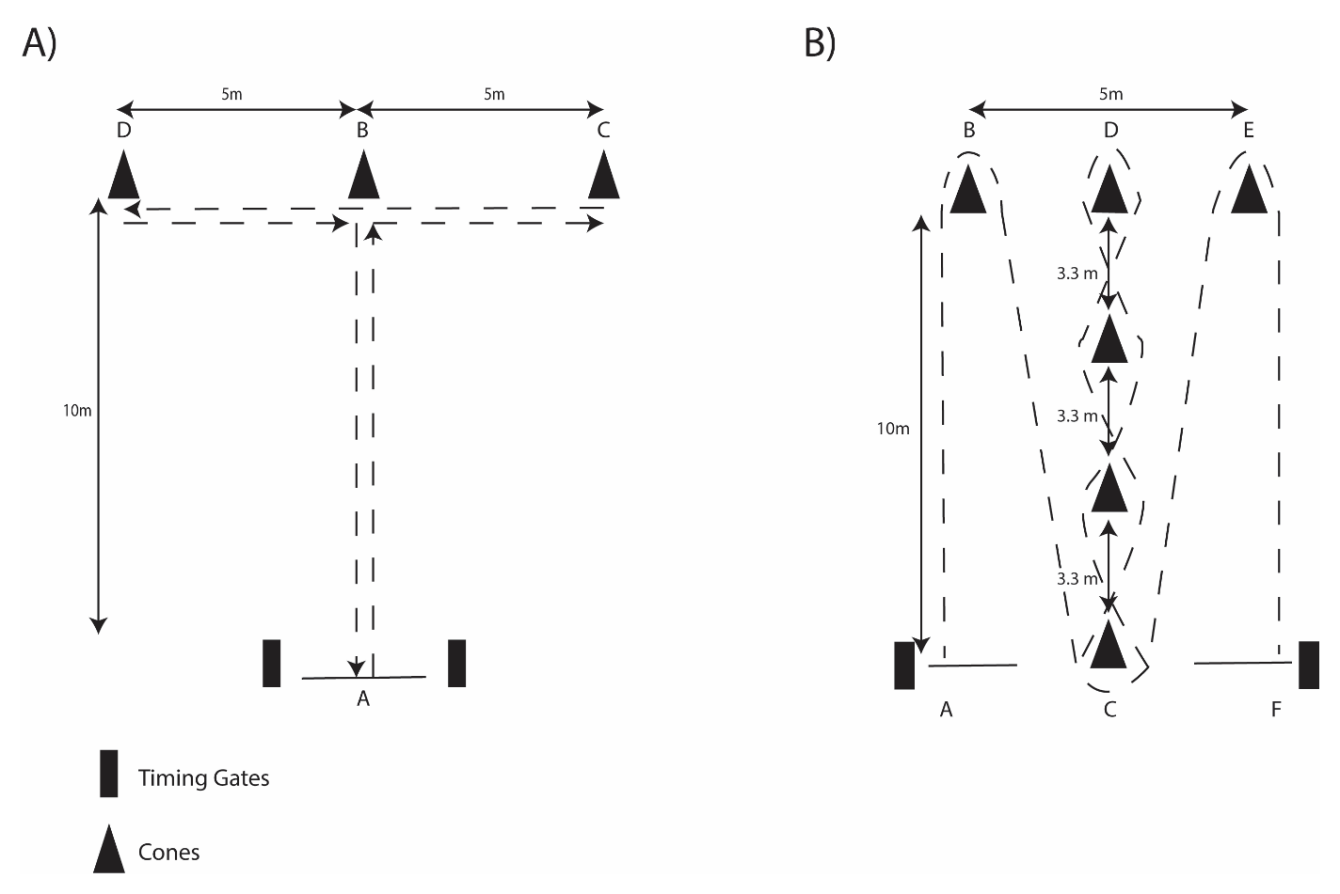
Schematic of **A**) T-Test Agility and **B**) Illinois Agility protocols.

Assessment of speed was achieved by completing 30-m maximal sprints. The Agility T-test protocol was set up within a 10-m × 10-m figure-T course ( Figure 2A) ^19^. The Illinois Agility protocol consisted of a 10-m × 5-m course ( Figure 2B) ^4, 20^. To ensure protocol familiarity, the participants completed three trials at sub-maximal effort followed by one final maximal trial, with each trial interspersed by two minutes of recovery. Trial completion times were recorded using an electronic timing gate system (Speedlight Timing Gates, Swift Performance, Australia) positioned at the start/finishing line with the same height and distances between the gates for each test and reported in seconds. The fastest time was used for later analysis.

### Repeat Sprint and Change-of-direction Protocols

The RSA, RTT and RIA protocols were completed by repeating the previously described protocols (i.e. 30-m sprint, T-test and Illinois Agility, respectively) across 6 repetitions with varying recovery periods in-between each repetition. Specifically, each repetition within the RSA, RTT and RIA was separated by 20-, 35- and 60-second recovery, respectively, with work-to-rest ratios of approximately 1:3 ^8, 9^. The participant’s instantaneous heart rate (HR, Polar Heart Rate Monitor, Polar H10, Finland) and rating of perceived exertion (RPE, Borg category scale 1-10) were collected at the completion of each repetition of the RSA, RTT and RIA protocols. The maximum and average HR and RPE values were then reported from the 6 repetitions ^21^. The following parameters were also calculated for each repeated agility protocol: total time (TT) of 6 cycles, best cycle time (BT), the average cycle time (AT) and fatigue index (FI) ^8^. FI was calculated as follows ^9^:

$$Fatigue\;Index=\left(\left(\frac{TT}{BT\times 6}\right)\times 100\right)-100$$

### Statistical analysis

Data was analysed using a statistical software (IBM SPSS version 25, Chicago, Illinois) and reported as mean ± standard deviation. Normality of the data was assessed using the Kolmogorov-Smirnov statistic. Construct validity of the repeated-COD protocols was identified via Pearson’s product moment correlation coefficients for RTT and RIA measures (i.e., TT, BT, AT and FI) and construct validity with aerobic capacity, leg power, speed and COD variables (i.e., VO _2max_, CMJ, 30-m sprint time, T-Test and Illinois Agility, respectively) were assessed as contributors to the RIA protocol. The cut-off for acceptable convergent validity and contributors to the RIA protocol was established when the association was statistically significant with an r-value of ≥ 0.70 ^22, 23^. Reliability of the repeated-COD measures was determined via a paired T-test, intraclass correlation coefficients (ICC, SPSS 2-way mixed, 95% confidence intervals), coefficient of variation (CV, 95% confidence intervals) and systematic bias/ratio with 95% limits of agreement (LOA) ^24^. Where significant relationships existed between the mean difference and average of test-retest values (i.e. heteroscedastic errors), variables were transformed (natural logarithm) prior to the calculation of measurement bias/ratio × / ÷ ratio LOA ^25^. The level of significance for all analyses was set at 0.05. Finally, effect size (Cohen’s *d*) with 95% CI was used to calculate the magnitude of differences in muscle soreness and CMJ measures between RIA and RTT protocols to determine whether the recovery periods were appropriate. The ES classifications were set as small, moderate and large with values of 0.2, 0.5 and 0.8, respectively (Cohen, 1988).

## Results

For convergent validity, significant correlations were identified between RIA and most RTT variables ( Table 1 ^26^). For contributors to RIA performance, significant correlations were identified with RSA, 30-m sprint time, best effort agility measures, aerobic capacity and CMJ height ( Table 2 ^26^).

**Table 1.** Relationship between performance measures of the repeated Illinois agility test (RIA), repeated T-agility test (RTT) and repeated sprint ability (RSA).

	RIA				RTT			
	TT (s)	BT (s)	AT (s)	FI (%)	TT (s)	BT (s)	AT (s)	FI (%)
RSA								
TT (s)	0.80 ***	0.73 ***	0.80 ***	0.55 ***	0.70 **	0.51 *	0.70 **	0.55 **
BT (s)	0.63 ***	0.51 ***	0.63 ***	0.56 ***	0.71 **	0.54 *	0.71 **	0.50 *
AT (s)	0.80 ***	0.73 ***	0.80 ***	0.55 ***	0.70 **	0.51 *	0.70 **	0.55 **
FI (%)	0.48 **	0.61 ***	0.49 **	0.28	0.28	0.13	0.28	0.37
RTT								
TT (s)	0.84 ***	0.81 ***	0.84 ***	0.51 ***	–	–	–	–
BT (s)	0.81 ***	0.81 ***	0.81 ***	0.44 ***	–	–	–	–
AT (s)	0.84 ***	0.80 ***	0.84 ***	0.51 ***	–	–	–	–
FI (%)	0.43 **	0.32	0.43 **	0.48 **	–	–	–	–

TT = total time; BT = best time; AT = average time; FI = fatigue index *P<0.05 **P<0.01 ***P<0.001

**Table 2.** Pearson correlation coefficients between repeated performances, perceptual and physiological indices (repeated Illinois agility [RIA], repeated T-agility test [RTT]) with aerobic capacity, leg power, speed, and agility test performance measures.

	VO _2max_ (mL·kg ^-1^·min ^-1^)	CMJ (cm)	Sprint 30m (sec)	IA (sec)	TTA (sec)
RIA					
TT (s)	-0.73 **	-0.85 **	0.89 **	0.87 **	0.72 **
BT (s)	-0.71 **	-0.79 **	0.81 **	0.86 **	0.71 **
AT (s)	-0.73 **	-0.85 **	0.89 **	0.87 **	0.72 **
FI (%)	-0.43 *	-0.57 **	0.61 **	0.49 *	0.40
HR _Avg_	-0.43	-0.09	0.34	0.24	0.38
HR _Max_	-0.20	-0.03	0.12	0.04	0.21
RPE _Avg_	0.17	0.17	-0.21	-0.16	-0.21
RPE _Max_	-0.04	-0.23	0.27	0.35	0.21
RTT					
TT (s)	-0.68 **	-0.76 **	0.80 **	0.84 **	0.80 **
BT (s)	-0.65 **	-0.74 **	0.80 **	0.86 **	0.85 **
AT (s)	-0.68 **	-0.76 **	0.80 **	0.84 **	0.80 **
FI (%)	-0.41	-0.37	0.34	0.28	0.14
HR _Avg_	-0.41	-0.05	0.26	0.13	0.23
HR _Max_	-0.33	-0.68	0.21	0.06	0.24
RPE _Avg_	0.00	0.02	-0.03	0.02	-0.07
RPE _Max_	0.02	0.04	-0.03	0.03	-0.03

CMJ = countermovement jump; TT= total time; BT = best time; AT = average time; FI = fatigue index ; Sprint 30m = 30 metre sprint; IA = Illinois Agility test; TTA = T-test agility; VO _2max_ = maximal aerobic capacity , RPE _Avg_= Average Rate of Perceived Exertion, RPE _Max_ = Maximum Rate of Perceived Exertion, HR _Avg_= Heart rate average, HR _Max_ = Maximum heart rate *P<0.05 **P<0.01 ***P<0.001

Most RIA measures exhibited excellent test-retest reliability (ICC = 0.92-0.97), good levels of agreement (ratio LOA = 1.05-1.06) and low measurement error (CV = 2.17 - 2.68%) ( Table 3) between Sessions 4 and 5. However, FI and average RPE demonstrated moderate test-retest reliability (ICC = 0.87 and 0.76, respectively), poorer levels of agreement (ratio LOA = 2.57 and 2.23, respectively) and higher measurement error (CV = 25.3 and 15.8%, respectively, Table 3).

For the RTT, excellent test-retest reliability (ICC = 0.91), good levels of agreement (ratio LOA = 1.08) and low measurement error (CV = 2.17-2.68%) were identified for TT, BT and AT ( Table 3) between Sessions 4 and 5, although maximum RPE demonstrated higher levels of measurement error (CV = 12.3%) ( Table 3). In addition, FI, average RPE and maximum HR displayed moderate to large reliability (ICC = 0.69 – 0.89), poorer agreement (ratio LOA = 1.10 – 2.59) and higher measurement error (CV = 2.38 – 27.6%) compared to the RIA protocol ( Table 3).

**Table 3.** Test-retest results, intra-class correlation coefficients (ICC, 95% confidence interval (CI)), measurement bias/ratio (log-transformed data) (×/÷ 95% ratio limits of agreement (ratio-LOA)) and within-subject coefficient of variation (95 % CI) of the repeated Illinois Agility (RIA) and T-test (RTT) protocol.

	Test (s)	Retest (s)	p	ICC (95% CI)	CV% (95% CI)	Bias ratio-LOA
RIA						
TT (s)	108.22 ± 9.14	107.38 ± 8.39	0.23	0.97 (0.92 - 0.99) ***	1.97 (0.91-2.16)	1.01 ×/ 1.06
BT (s)	17.00 ± 1.03	17.05 ± 1.05	0.60	0.96 (0.90 - 0.98) ***	1.77 (0.98-1.83)	1.00 ×/ 1.05
AT (s)	18.04 ± 1.52	17.90 ± 1.40	0.23	0.97 (0.92 - 0.99) ***	1.97 (0.91-2.16)	1.01 ×/ 1.06
FI (%)	6.02 ± 3.50	4.91 ± 3.18	0.03 †	0.87 (0.68 - 0.95) ***	25.3 (22.9-40.1)	1.32 ×/ 2.57
RPE _Avg_	4.9 ± 1.2	4.3 ± 1.7	0.07	0.76 (0.41-0.90) **	15.8 (6.1-25.6)	1.20 ×/ 2.23
RPE _Max_	6.5 ± 1.6	6.1 ± 1.7	0.04 †	0.93 (0.83-0.97) ***	8.1 (4.8-11.7)	1.08 ×/ 1.34
HR _Avg_ (bpm)	183.8 ± 8.5	180.2 ± 10.2	0.09	0.92 (0.89-0.97) ***	2.10 (1.48-2.72)	1.02 ×/ 1.06
HR _Max_ (bpm)	189.0 ± 8.3	188.3 ± 9.6	0.53	0.94 (0.83-0.98) ***	1.31 (0.78-1.84)	1.00 ×/ 1.05
RTT						
TT (s)	68.69 ± 4.79	69.01 ± 5.15	0.61	0.91 (0.79 - 0.96) ***	2.68 (1.91-1.39)	1.00 ×/ 1.08
BT (s)	11.01 ± 0.7	11.06 ± 0.74	0.58	0.91 (0.78 - 0.96) ***	2.17 (1.55-2.80)	1.00 ×/ 1.08
AT (s)	11.45 ± 0.80	11.50 ± 0.86	0.61	0.91 (0.79 - 0.96) ***	2.68 (1.91-3.14)	1.00 ×/ 1.08
FI (%)	3.98 ± 1.68	3.97 ± 1.89	0.99	0.69 (0.25 - 0.87) **	27.6 (15.3-33.7)	1.03 ×/ 2.59
RPE _Avg_	3.2 ± 1.2	3.6 ± 1.4	0.10	0.89 (0.73-0.95) ***	15.3 (8.4-22.2)	0.91 ×/ 1.85
RPE _Max_	4.4 ± 1.7	4.9 ± 1.8	0.02 †	0.93 (0.84-0.97) ***	12.3 (4.7-19.8)	0.90 ×/ 1.78
HR _Avg_ (bpm)	176.2 ± 7.5	174.0 ± 13.0	0.35	0.79 (0.45-0.92) **	2.88 (1.70-4.04)	1.02 ×/ 1.11
HR _Max_ (bpm)	186.3 ± 8.0	183.7 ± 10.0	0.20	0.68 (0.14-0.88) *	2.38 (1.06-3.69)	1.02 ×/ 1.10

TT= total time; BT = best time; AT = average time; FI = fatigue index, RPE _Avg_ = Average Rate of Perceived Exertion, RPE _Max_ = Maximum Rate of Perceived Exertion, HR _Avg_ = Heart rate average, HR _Max_ = Maximum heart rate P<0.05 **P<0.01 *** P<0.001

† Significantly different (p<0.05)

No significant differences were found for muscle soreness (p = 0.10) and CMJ performance (p = 0.80) between the testing sessions.

## Discussion

This study showed that the RIA and RTT protocols were strongly correlated with each other, particularly with respect to the time-derived measures (BT, TT and AT). In addition, strong correlations were identified between the time-derived measures of RIA with VO2max, CMJ and 30-m sprint performance. Excellent test-retest reliability was evident for the time-derived, perceptual and physiological measures of the RIA protocol, although FI was questionable. The current findings support RIA is a reliable and valid assessment of COD and fitness in young RL players.

The strong correlations of the time-derived measures (BT, TT and AT) between the RIA and RTT protocols, highlighted the RIA protocol was a valid assessment of a repeated-COD, but with movement demands more representative of RL. In addition, the TT and BT of the RIA was strongly associated with the TT and BT of the RSA, indicating that the ability to maintain linear speed would result in superior performances in the RIA protocol, possibly due to similar metabolic demands ^7^. Comparable findings were reported by Fessi, Makni ^9^, with strong correlations identified between the BT and TT of their repeated agility protocol and RSA protocols in 45 team-sport athletes. The comparable measures between RIA, RTT and RSA suggests that anaerobic fitness, in conjunction with efficient recovery dynamics during short periods of rest in-between explosive activities, are essential qualities for optimal performance in an RIA protocol. Collectively, our results and others ^7, 9^, suggest that performance of repeated-COD relies heavily upon the anaerobic system, a metabolic pathway predominant in RL ^27^.

The current study also identified strong test-retest reliability for time-derived measures (i.e., BT, TT and AT) of the RIA, with minimal measurement error. However, the measurement error was substantially higher for FI, confirming previous studies that reported substantially stronger reliability measures for BT, TT and AT compared to that of FI from various repeated-COD protocols ^7, 8, 28^. It has been suggested that FI may exhibit weaker reproducibility as the measure is multifactorial and dependent on the stability of other variables (i.e., TT and BT) ^7, 29^. Subsequently, we, and others ^7, 8, 28, 29^, recommend that time-derived measures be primarily evaluated during repeated-COD protocols.

Another novelty of the current study was the reliability of the psychophysiological responses during both RIA and RTT protocols. The test-retest reliability values for HR and RPE ranged between questionable-to-excellent classifications according to ICC scores for both RIA and RTT. However, distinctly greater measurement error and bias was observed for RPE when compared to HR measures for both RIA and RTT. These findings were similar to previous studies with poorer reliability for RPE than HR measures during various running protocols ^30– 32^. It has been postulated that HR has better stability across days given that it is an objective measure, compared to the highly subjective RPE ^33^. It has also been reported that participant’s prior knowledge of the number of sprints during repeated sprint-type protocols may affect results due to pacing ^34^. Accordingly, HR measures may be a better physiological indicator for monitoring exercise-induced stress during repeated-COD protocols.

An additional, yet essential finding of this study was the relationship between baseline characteristics and performances measures from the repeated-COD tests. Measures of CMJ, best-effort speed and best-effort COD performance correlated significantly with the time-derived variables of the RIA. These relationships indicated that lower limb power, linear speed and COD capabilities were contributing factors to successful repeated-COD performances. Our findings aligned with those of Haj-Sassi, Dardouri ^8^, who reported strong correlations between measures of jump performance and repeated-COD performance with an Agility T-test protocol. Similar findings were also reported by previous studies with muscular strength, and linear and COD speed considered strong contributors of COD performance ^35, 36^. The significance of this finding attests to lower limb power production being a critical component of repeated-COD performance, especially within the RIA.

Finally, the current study identified significant correlations between VO _2max_ and RIA performance measures. These findings are similar to previous studies using various repeated-COD protocols ^28, 29^ as well as RSA protocols ^37– 39^. Measures of VO _2max_ has been considered essential for repeated-sprint type protocols, due to muscular reoxygenation rate ^8, 40^, optimal capacity to remove and buffer hydrogen ions within working muscles ^41^ and efficiently replenish phosphagen stores ^42^. The findings of the present study suggest that aerobic capacity is a strong contributor to superior repeated-COD efforts, further highlighting the need to optimise recovery capacities between high-intensity bouts for RL athletes.

In conclusion, the RIA protocol exhibited moderate-to-excellent test-retest reliability and low measurement error for the majority of time-derived measures and psychophysiological measures, and questionable reliability for FI. Further, the RIA protocol showed strong correlations with the RTT protocol, demonstrating that the RIA protocol provided a valid measure of repeated COD performance. Finally, this study has clearly demonstrated that repeated agility performances rely upon contributions from both anaerobic and aerobic systems with the RIA, demonstrating that the qualities required for optimal RIA performance may be representative of the physical demands in RL. The RIA protocol may provide practitioners with a simple, yet effective monitoring tool to quantify athlete’s ability to generate and sustain multi-directional efforts, and their ability to recover during intermittent activities.

## Data availability

### Underlying data

James Cook University Research Data: Convergent validity and reliability of a novel repeated agility protocol in junior rugby league players. https://doi.org/10.25903/5eb0f568fad20 ^26^

This project contains the following underlying data:

- Raw_data_De-identified.xlsx (Agility protocol data in excel format)
- Raw_data_De-identified.ods (Agility protocol data in ods format)

Data are available under the terms of the Creative Commons Attribution 4.0 International license (CC-BY 4.0).
