# Supplementary material for: Convergent validity and reliability of a novel repeated agility protocol in junior rugby league players
Source: F1000Res. 2021 Nov 22;9:624. Originally published 2020 Jun 17. [Version 3] doi: 10.12688/f1000research.23129.3 (PMC8577058; doi:10.12688/f1000research.23129.3)
Supplement: Peer review history

# Peer review: Reviewer response for version 3

**DOI:** 10.5256/f1000research.79608.r100796

**Published:** 2021-11-22

**Authors:** Aaron T. Scanlan

**Affiliations:** 1 Human Exercise and Training Laboratory, School of Health, Medical and Applied Sciences, Central Queensland University, Rockhampton, Qld, Australia

Thank you for making the requested changes, I have no further comments to add.

Is the work clearly and accurately presented and does it cite the current literature?

Yes

If applicable, is the statistical analysis and its interpretation appropriate?

Yes

Are all the source data underlying the results available to ensure full reproducibility?

Yes

Is the study design appropriate and is the work technically sound?

Partly

Are the conclusions drawn adequately supported by the results?

Yes

Are sufficient details of methods and analysis provided to allow replication by others?

Yes

Reviewer Expertise:

Exercise and sport science

I confirm that I have read this submission and believe that I have an appropriate level of expertise to confirm that it is of an acceptable scientific standard.

# Peer review: Reviewer response for version 2

**DOI:** 10.5256/f1000research.78004.r96566

**Published:** 2021-11-08

**Authors:** James Dugdale

**Affiliations:** 1 School of Applied Sciences, University of Stirling, Stirling, UK; 2 Edinburgh Napier University, Edinburgh, UK

Thank you to the authors for their time and efforts taken to revise this manuscript. I am of the opinion that these revisions have strengthened the manuscript substantially.

 I am happy to approve the manuscript in its current form.

Is the work clearly and accurately presented and does it cite the current literature?

Partly

If applicable, is the statistical analysis and its interpretation appropriate?

Yes

Are all the source data underlying the results available to ensure full reproducibility?

Yes

Is the study design appropriate and is the work technically sound?

Yes

Are the conclusions drawn adequately supported by the results?

Yes

Are sufficient details of methods and analysis provided to allow replication by others?

Yes

Reviewer Expertise:

Fitness testing; youth athletes; talent identification; talent development; physical performance; strength and conditioning

I confirm that I have read this submission and believe that I have an appropriate level of expertise to confirm that it is of an acceptable scientific standard.

# Response

**Published:** 2021-11-18

**Authors:** Kenji Doma

**Affiliations:** James Cook University, Australia

Thank you very much for your comments.

# Peer review: Reviewer response for version 2

**DOI:** 10.5256/f1000research.78004.r96565

**Published:** 2021-10-11

**Authors:** Aaron T. Scanlan

**Affiliations:** 1 Human Exercise and Training Laboratory, School of Health, Medical and Applied Sciences, Central Queensland University, Rockhampton, Qld, Australia

Thank you for the opportunity to further review this submission. I thank the authors for giving such extensive consideration to the previous reviewer suggestions and making relevant changes where applicable. The manuscript has been strengthened, and I only have some further minor comments for consideration:

1. Abstract - thank you for adding "Beep test", but for consistency with the article text, consider changing this to "Multistage Shuttle test".
2. Introduction, paragraph 4, second aim - is RSA meant to be aerobic capacity here? In either case, aerobic capacity needs to be included in this aim specifically alongside the other fitness attributes.
3. Methods, 30-m sprint and agility protocols - be sure to check headings as well given previous suggestions on use of the term "agility". Also in this section, please indicate if the position of the timing gates were kept consistent across all participants with mention of the height (above ground) and distance apart available.
4. Statistical analysis - still use of "construct validity" on the fourth line in this paragraph as per previous revisions.
5. Discussion - there is a random equals sign on the last line of the fifth paragraph.

Is the work clearly and accurately presented and does it cite the current literature?

Yes

If applicable, is the statistical analysis and its interpretation appropriate?

Yes

Are all the source data underlying the results available to ensure full reproducibility?

Yes

Is the study design appropriate and is the work technically sound?

Partly

Are the conclusions drawn adequately supported by the results?

Yes

Are sufficient details of methods and analysis provided to allow replication by others?

Yes

Reviewer Expertise:

Exercise and sport science

I confirm that I have read this submission and believe that I have an appropriate level of expertise to confirm that it is of an acceptable scientific standard.

# Response

**Published:** 2021-11-18

**Authors:** Kenji Doma

**Affiliations:** James Cook University, Australia

Reviewer’s comment: Thank you for the opportunity to further review this submission. I thank the authors for giving such extensive consideration to the previous reviewer suggestions and making relevant changes where applicable. The manuscript has been strengthened, and I only have some further minor comments for consideration:

 Author’s response: Thank you very much for your comments, we believe your input has strengthened the quality of our work.

 Reviewer’s comment: Abstract - thank you for adding "Beep test", but for consistency with the article text, consider changing this to "Multistage Shuttle test".

 Author’s response: Amended as requested.

 Reviewer’s comment: Introduction, paragraph 4, second aim - is RSA meant to be aerobic capacity here? In either case, aerobic capacity needs to be included in this aim specifically alongside the other fitness attributes.

 Author’s response: This is a great pickup, now amended to aerobic capacity.

 Reviewer’s comment: Methods, 30-m sprint and agility protocols - be sure to check headings as well given previous suggestions on use of the term "agility". Also in this section, please indicate if the position of the timing gates were kept consistent across all participants with mention of the height (above ground) and distance apart available.

 Author’s response: The headings have now been changed to change-of-direction, and the height and position of the gates were described as being consistent for each test.

 Reviewer’s comment: Statistical analysis - still use of "construct validity" on the fourth line in this paragraph as per previous revisions.

 Author’s response: Changed to construct validity.

 Reviewer’s comment: Discussion - there is a random equals sign on the last line of the fifth paragraph.

 Author’s response: Thank you, this is now amended.

# Peer review: Reviewer response for version 1

**DOI:** 10.5256/f1000research.25532.r90678

**Published:** 2021-08-03

**Authors:** Aaron T. Scanlan

**Affiliations:** 1 Human Exercise and Training Laboratory, School of Health, Medical and Applied Sciences, Central Queensland University, Rockhampton, Qld, Australia

Thank you for the opportunity to review this manuscript. A logical study with potential practical impact was presented exploring the validity and reliability of a repeated change-of-direction speed test suited to rugby league. This aspect of fitness testing is lacking in the literature and of use for end-users, so this manuscript has merit for publication. I do however have some suggestions and queries listed below that will help strengthen some aspects of the work that should be addressed by the authors:

1. Abstract, Methods – indicate whether you are referring to the multi-stage fitness test (i.e., Beep test) here or if just an assessment of multi-stage fitness was performed.
2. Abstract – some justification of using the repeated T-agility test as the other standard repeated change-of-direction performance to assess convergent agility of the repeated Illinois Agility test is needed in the Abstract.
3. Abstract – the focus of exploring the contribution of different fitness attributes to repeated Illinois Agility test performance is not made in the Abstract. Consider including this as a secondary objective and/or making it clear why this analysis is needed.
4. Abstract, Conclusions – make it clear that this test is useful for Rugby League coaches specifically, and change “athlete” to plural form or indicate “team” here instead.
5. Introduction, opening paragraph – in this paragraph you identify “agility” and hint that it involves physical and cognitive components. So in essence, you are examining change-of-direction speed (the physical component) rather than “agility” per se. Consider making this clear and using the term “change-of-direction speed” or “change-of-direction performance” thereafter when referring to tests and attributes that are purely physical without the cognitive component.
6. Introduction, opening paragraph – stronger rationale is needed justifying the inclusion of the Agility T-test and Illinois Agility test in rugby league. Can you add a sentence or two outlining why these tests are suited to the sport and therefore the focus of your study?
7. Introduction, end of second paragraph – here indicate whether the validity and reliability of this test has not been investigated at all or just specifically in Rugby League athletes. Also, at the end of this sentence make it clear that you are referring to the usability of this test.
8. Introduction, aims – for the second aim, you are not comparing the RIA measures to other measures, but instead correlating them, so please change this aim accordingly. Also, consider including a little rationale around this aim in the previous paragraph as it is unclear as to why this is important.
9. Introduction, aims – it might pay to include a sentence stating why examining convergent validity (e.g. to show that you are assessing similar attributes with a new test that is more practical and specific to Rugby League when compared to a standard, generic test routinely used) and retest reliability (e.g. to detect the inherent error in the test and ascertain whether it can be reliably adopted in practice to assess repeated measurements in athletes) are needed for practical uptake of the test, which would strengthen the rationale of the first and third aims as well in the Introduction section.
10. Introduction, hypotheses – you only mention speed and anaerobic capacity as your fitness attributes here, but you also included aerobic fitness (Multistage Fitness test)?
11. Methods, Research design – why was the order flipped between session 4 and session 5? It seems like you would want to the athletes to complete the same exact session across both when assessing retest reliability as it is introducing a confounding factor? Also, no hyphen needed between “15” and “minutes” here.
12. Methods, Participants – can you provide some further indication as to the specific playing level of the athletes? The name of the program is great, but this is not exactly clear for all readers.
13. Figure 1 – consider changing the session numbers, as in text you identify the Multistage Fitness test as session 1, but here you indicate the other fitness testing as session 1. Consistency needed.
14. Methods, Participants – add “in” after “partaking” and make parents/guardians singular rather than plural.
15. Methods, Multistage Shuttle test – stay consistent with capitalising the test names like this one as it is done inconsistently throughout. Also, hyphenate “20m” here.
16. Methods, Countermovement jump test – hyphenate “1 cm” here. Also, try to indicate what units the key outcomes from each test were reported in (e.g. mL/kg/min, cm, s).
17. Methods, 30-m Sprint and Agility protocols – separate statements on linear and change-of-direction speed here. Also, hyphenate “10m” and “5m” for the Illinois Agility test.
18. Methods, repeat protocols – to calculate average HR and RPE, was HR measured from when the test started to when the test finished in 1-second intervals? And was RPE taken after each effort or just after all efforts for each specific test? These are not quite clear.
19. Methods, Statistical analysis – you mention “construct” validity for the first time here. If this is a key aim and aspect of the study (i.e., correlating performance during the test with fitness attributes), then this needs to be established earlier (i.e., introduction and aims).
20. Methods, Statistical analysis – an r value of 0.5 seems quite low to establish convergent validity (only 25% shared variance)? In this regard, what was the cut-off for construct validity?
21. Results, first paragraph – clarify whether this is CMJ height specifically.
22. Results, second paragraph – make it clear that you are referring to the third and fourth sessions here (and make sure this is consistent as you identify these as the fourth and fifth sessions earlier).
23. Results, third paragraph – you do not indicate the maximum RPE was taken as an outcome in the methods anywhere (only average), yet it is listed here? Same for maximum HR, which appears later in this section also.
24. Table 1 – shouldn’t RIA and RTT be the two key tests going from left to right rather than RIA and RSA? In fact, it is not clear why RSA is included here given you identify RIA vs. RTT for convergent validity assessment throughout the manuscript. Please adjust or rework earlier sections.
25. Discussion, first paragraph – some minor errors, but change “was” to “were” in the 7 ^th^ line.
26. Discussion, first paragraph – at the end of this paragraph, some explanation as to how yours (and the previous studies) support that anaerobic fitness is predominantly stressed in the repeated COD tests. I am assuming the strong correlations with mostly anaerobic fitness attributes, but this is an assumption and should be explained further to clarify this statement.
27. Discussion, fourth paragraph – be careful with your statement suggesting they are “key attributes for RL athletes”. You did not show this but state it, so rework or remove this part to focus specifically on what your data show.
28. Discussion, concluding paragraph – here you highlight how anaerobic and aerobic fitness underpin test performance, but previously you focus on anaerobic fitness in other sections and in this section. Please make sure the message is consistent throughout.

Is the work clearly and accurately presented and does it cite the current literature?

Yes

If applicable, is the statistical analysis and its interpretation appropriate?

Yes

Are all the source data underlying the results available to ensure full reproducibility?

Yes

Is the study design appropriate and is the work technically sound?

Partly

Are the conclusions drawn adequately supported by the results?

Yes

Are sufficient details of methods and analysis provided to allow replication by others?

Yes

Reviewer Expertise:

Exercise and sport science

I confirm that I have read this submission and believe that I have an appropriate level of expertise to confirm that it is of an acceptable scientific standard, however I have significant reservations, as outlined above.

# Response

**Published:** 2021-09-27

**Authors:** Kenji Doma

**Affiliations:** James Cook University, Australia

Reviewer’s comment: Thank you for the opportunity to review this manuscript. A logical study with potential practical impact was presented exploring the validity and reliability of a repeated change-of-direction speed test suited to rugby league. This aspect of fitness testing is lacking in the literature and of use for end-users, so this manuscript has merit for publication. I do however have some suggestions and queries listed below that will help strengthen some aspects of the work that should be addressed by the authors:

**Author’s response: Dear Reviewer, thank you very much for your comments. We believe your feedback has improved the quality of our work.**

 Reviewer’s comment: Abstract, Methods – indicate whether you are referring to the multi-stage fitness test (i.e., Beep test) here or if just an assessment of multi-stage fitness was performed.

**Author’s response: A Beep test was performed, which is now included in the Abstract.**

 Reviewer’s comment: Abstract – some justification of using the repeated T-agility test as the other standard repeated change-of-direction performance to assess convergent agility of the repeated Illinois Agility test is needed in the Abstract.

**Author’s response: This is a valid point. The Background now includes, “Thus, the current study examined the convergent validity of a repeated Illinois Agility (RIA) protocol with the repeated T-agility protocol, and the repeatability of the RIA protocol in adolescent Rugby League players.”**

 Reviewer’s comment: Abstract – the focus of exploring the contribution of different fitness attributes to repeated Illinois Agility test performance is not made in the Abstract. Consider including this as a secondary objective and/or making it clear why this analysis is needed.

**Author’s response: Thank you for the suggestion. We have now included, “Furthermore, aerobic capacity and anaerobic and COD performance were assessed to determine whether these physical qualities were important contributors to the RIA protocol.”**

 Reviewer’s comment: Abstract, Conclusions – make it clear that this test is useful for Rugby League coaches specifically, and change “athlete” to plural form or indicate “team” here instead.

**Author’s response: The conclusion has been rewritten to, “The current study has demonstrated the RIA to be a simple, valid and reliable field test for RL athletes that can provide coaches with information about their team’s ability to sustain high intensity, multi-directional running efforts.”**

 Reviewer’s comment: Introduction, opening paragraph – in this paragraph you identify “agility” and hint that it involves physical and cognitive components. So in essence, you are examining change-of-direction speed (the physical component) rather than “agility” per se. Consider making this clear and using the term “change-of-direction speed” or “change-of-direction performance” thereafter when referring to tests and attributes that are purely physical without the cognitive component.

**Author’s response: All the terms with ‘agility’ has been replaced with ‘change-of-direction’ performance or protocols throughout the text.**

 Reviewer’s comment: Introduction, opening paragraph – stronger rationale is needed justifying the inclusion of the Agility T-test and Illinois Agility test in rugby league. Can you add a sentence or two outlining why these tests are suited to the sport and therefore the focus of your study?

**Author’s response: We have removed Agility T-test and Illinois Agility test from the first paragraph, and focused the justification of the Illinois Agility test for RL in the second paragraph (see lines 41-47).**

 Reviewer’s comment: Introduction, end of second paragraph – here indicate whether the validity and reliability of this test has not been investigated at all or just specifically in Rugby League athletes. Also, at the end of this sentence make it clear that you are referring to the usability of this test.

**Author’s response: We separated the second paragraph into two paragraphs. Thus, we highlighted that no studies have examined the validity and reliability of the RIA protocol at the end of the fourth paragraph.**

 Reviewer’s comment: Introduction, aims – for the second aim, you are not comparing the RIA measures to other measures, but instead correlating them, so please change this aim accordingly. Also, consider including a little rationale around this aim in the previous paragraph as it is unclear as to why this is important.

**Author’s response: Changed to ‘correlating’ as requested. The previous paragraph expanded on the need to examine the validity of the RIA protocol, which we now hope provides further justification of our aims.**

****

 Reviewer’s comment: Introduction, aims – it might pay to include a sentence stating why examining convergent validity (e.g. to show that you are assessing similar attributes with a new test that is more practical and specific to Rugby League when compared to a standard, generic test routinely used) and retest reliability (e.g. to detect the inherent error in the test and ascertain whether it can be reliably adopted in practice to assess repeated measurements in athletes) are needed for practical uptake of the test, which would strengthen the rationale of the first and third aims as well in the Introduction section.

**Author’s response: This is a great suggestion. We have now worded the end of the Introduction to, “Examining the convergent validity of the RIA protocol will determine whether this novel assessment exhibits similar attributes to a standardised COD protocol (i.e., RTT). In addition, the reliability of the RIA will determine whether this test can be reliably adopted in practice by accounting for the inherent error of the test across repeated measurements. The quality of these psychometric properties will provide coaches with a tool to assist in monitoring and training RL athletes as well as in talent development and identification.”**

 Reviewer’s comment: Introduction, hypotheses – you only mention speed and anaerobic capacity as your fitness attributes here, but you also included aerobic fitness (Multistage Fitness test)?

**Author’s response: Included aerobic capacity in the hypotheses.**

 Reviewer’s comment: Methods, Research design – why was the order flipped between session 4 and session 5? It seems like you would want to the athletes to complete the same exact session across both when assessing retest reliability as it is introducing a confounding factor? Also, no hyphen needed between “15” and “minutes” here.

**Author’s response: This is a good question. We employed a cross-over design for sessions 4 and 5 to limit the confounder of any learning effects (i.e., were participants still able to exhibit reproducible measures when the protocols were swapped around, as opposed to the participants becoming accustomed to the order effects between sessions). We have also included the hyphen.**

 Reviewer’s comment: Methods, Participants – can you provide some further indication as to the specific playing level of the athletes? The name of the program is great, but this is not exactly clear for all readers.

**Author’s response: Further description of the excellence program has been provided.**

 Reviewer’s comment: Figure 1 – consider changing the session numbers, as in text you identify the Multistage Fitness test as session 1, but here you indicate the other fitness testing as session 1. Consistency needed.

**Author’s response: Session 1 included in the Multistage Fitness test as requested (see updated Figure 1).**

 Reviewer’s comment: Methods, Participants – add “in” after “partaking” and make parents/guardians singular rather than plural.

**Author’s response: Amended as requested.**

 Reviewer’s comment: Methods, Multistage Shuttle test – stay consistent with capitalising the test names like this one as it is done inconsistently throughout. Also, hyphenate “20m” here.

**Author’s response: Amended the test name to upper case as requested and hyphenated to 20-m.**

 Reviewer’s comment: Methods, Countermovement jump test – hyphenate “1 cm” here. Also, try to indicate what units the key outcomes from each test were reported in (e.g. mL/kg/min, cm, s).

**Author’s response: Hyphenated to 1-cm and reported units of measure as requested for each test.**

 Reviewer’s comment: Methods, 30-m Sprint and Agility protocols – separate statements on linear and change-of-direction speed here. Also, hyphenate “10m” and “5m” for the Illinois Agility test.

**Author’s response: Statements have been separated and hyphenated as requested.**

****

 Reviewer’s comment: Methods, repeat protocols – to calculate average HR and RPE, was HR measured from when the test started to when the test finished in 1-second intervals? And was RPE taken after each effort or just after all efforts for each specific test? These are not quite clear.

**Author’s response: We have included “ “The participant’s instantaneous heart rate (HR, Polar Heart Rate Monitor, Polar H10, Finland) and rating of perceived-exertion (RPE, Borg category scale 1-10) were collected at the completion of each repetition of the RSA, RTT and RIA protocols. The maximum and average HR and RPE values were then reported from the 6 repetitions. – see lines 138-141.**

 Reviewer’s comment: Methods, Statistical analysis – you mention “construct” validity for the first time here. If this is a key aim and aspect of the study (i.e., correlating performance during the test with fitness attributes), then this needs to be established earlier (i.e., introduction and aims).

**Author’s response: We have taken out “construct validity” and mentioned aerobic capacity, power and COD as “contributors to the RIA protocol”.**

 Reviewer’s comment: Methods, Statistical analysis – an r value of 0.5 seems quite low to establish convergent validity (only 25% shared variance)? In this regard, what was the cut-off for construct validity?

**Author’s response: This has been re-established to a minimum r-value of 0.7 for both convergent validity and contributors to the RIA protocol.**

 Reviewer’s comment: Results, first paragraph – clarify whether this is CMJ height specifically.

**Author’s response: Height included as requested.**

 Reviewer’s comment: Results, second paragraph – make it clear that you are referring to the third and fourth sessions here (and make sure this is consistent as you identify these as the fourth and fifth sessions earlier).

**Author’s response: Sessions numbers included as requested, and we also applied this approach in the next paragraph for the RTT protocol to ensure clarity.**

****

 Reviewer’s comment: Results, third paragraph – you do not indicate the maximum RPE was taken as an outcome in the methods anywhere (only average), yet it is listed here? Same for maximum HR, which appears later in this section also.

**Author’s response: This is a great pick up. In the methods section, we have now included, “The participant’s heart rate (HR, Polar Heart Rate Monitor, Polar H10, Finland) and rating of perceived-exertion (RPE, Borg category scale 1-10) were collected at the completion of each repetition of the RSA, RTT and RIA protocols. The maximum and average HR and RPE values were then reported from the 6 repetitions.”**

 Reviewer’s comment: Table 1 – shouldn’t RIA and RTT be the two key tests going from left to right rather than RIA and RSA? In fact, it is not clear why RSA is included here given you identify RIA vs. RTT for convergent validity assessment throughout the manuscript. Please adjust or rework earlier sections.

**Author’s response: Table 1 has now been amended as requested. RSA was included more to determine the importance of the athlete’s ability to sustain speed and effort over repeated bouts, which has now been included in the second aim.**

 Reviewer’s comment: Discussion, first paragraph – some minor errors, but change “was” to “were” in the 7th line.

**Author’s response: Amended as requested.**

****

 Reviewer’s comment: Discussion, first paragraph – at the end of this paragraph, some explanation as to how yours (and the previous studies) support that anaerobic fitness is predominantly stressed in the repeated COD tests. I am assuming the strong correlations with mostly anaerobic fitness attributes, but this is an assumption and should be explained further to clarify this statement.

**Author’s response: Closer to the end of this paragraph, we have included a sentence that reads, “The comparable measures between RIA, RTT and RSA suggests that anaerobic fitness, in conjunction with efficient recovery dynamics during short periods of rest in-between explosive activities, are essential qualities for optimal performance in an RIA protocol.”**

****

 Reviewer’s comment: Discussion, fourth paragraph – be careful with your statement suggesting they are “key attributes for RL athletes”. You did not show this but state it, so rework or remove this part to focus specifically on what your data show.

**Author’s response: Removed as requested.**

 Reviewer’s comment: Discussion, concluding paragraph – here you highlight how anaerobic and aerobic fitness underpin test performance, but previously you focus on anaerobic fitness in other sections and in this section. Please make sure the message is consistent throughout.

**Author’s response: We have now included aerobic capacity as possible important contributors to the RIA protocol in the Abstract, Introduction (aim and hypothesis) and earlier sections in the Discussion.**

# Peer review: Reviewer response for version 1

**DOI:** 10.5256/f1000research.25532.r74005

**Published:** 2020-11-09

**Authors:** James Dugdale

**Affiliations:** 1 School of Applied Sciences, University of Stirling, Stirling, UK; 2 Edinburgh Napier University, Edinburgh, UK

The present study aimed to explore the validity and reliability of repeated “agility” tests in a sample of youth rugby players. The study provides interesting data for an applied audience and is methodologically sound and accurate in the presentation of findings. This being said, I have identified a range of areas that should be improved prior to potential resubmissions of this manuscript. For example, the introduction (and study in its entirety) is inaccurate in its use of the term “agility” (opposed to COD which the authors actually assess). Furthermore, the introduction needs to be further developed to support the scope of the study. The authors need to create more of a rationale for running such a large number of correlations – exploring correlational analyses needs to be warranted based upon an assumption of a relationship (or lack thereof) between variables. In its current form, I am unsure whether this manuscript provides such rationale. The methods and results are accurate, but with a large number of small errors. Finally, the discussion addresses key discussion points formulated from the findings of the study, but similar to the introduction, lacks some critical depth.

 I do think that the authors have a publishable study here, however, a number of revisions will be required before I would be happy to approve this manuscript for indexing.

 I hope that my comments are received as I intend them and are useful in further strengthening this manuscript. I hope to receive the opportunity to review this manuscript again in its revised form.

**Abstract**

 The abstract is concise and accurate to the details of the study. I have just a couple of minor comments (below):

 The initial sentence is accurate and outlines the requirement of the study. However, I would encourage the authors to insert a word such as ‘simultaneously’ or ‘congruently’, or attempt to capture the lack of a suitable test to capture the performance of these abilities in combination.

 Abstract Methods – the inclusion of the repeated T-agility test appears suddenly with no prior reference. If this is a key concentration of your study, I would encourage to insert earlier in the abstract along with the RIA.

 Abstract Results – please specify exact p values rather than just <0.05. CV% can be presented to 1dp for brevity.

**Introduction**

 The introduction is short and concise. However, I feel some key information that would further support the rationale for your study has been omitted. I would encourage the authors to further develop this section. A suggestion would be to expand the second paragraph into two: the first discussing the validity aspects, the second the reliability aspects. I do not expect these changes to be too onerous, but believe it would strengthen this section of your manuscript if you were to implement these changes. I have some further specific comments below.

 The opening sentence would read better if it were split into two separate sentences.

 The authors should attempt to clarify during opening paragraph that they are discussing change of direction (COD) performance, rather than agility. Although COD performance is a key component of agility performance, the lack of an external stimuli prevents classification of agility. Although the tests identified by the authors in this opening paragraph have titled themselves ‘agility’ tests, by definition they are not. The authors should be careful in their stance here as the literature has evolved substantially since the inception of tests such as these, and appropriate classification and terminology is essential for the present study.

 Young,  W.;   Dawson,  B.;   Henry,  G.  Agility  and  Change-of-Direction  Speed  are  Independent  Skills: Implications for Agility in Invasion Sports. Int. J. Sports Sci. Coach. 2015, 10, 159–169. ^1^ 

 Nimphius,  S.;   Callaghan,  S.J.;   Bezodis,  N.E.;   Lockie,  R.G.  Change  of  Direction  and  Agility  Tests: Challenging Our Current Measures of Performance. Strength Cond. J. 2018, 40, 26–38. ^2^

 Sheppard, J.M.; Young, W.B. Agility literature review: Classifications, training and testing. J. Sports Sci. 2006, 24, 919–932. ^3^

 Regarding the reliability aspect of the study, two recent papers have suggested that reliability of COD and agility may be lower in adolescent and youth athletes (as per your sample). Although these authors observed this during maximal COD/agility performance, opposed to repeated as in the present study, I believe this would be worth including and would further warrant the exploration of your research question and study.

 Taylor, J.M.; Cunningham, L.; Hood, P.; Thorne, B.; Irvin, G.; Weston, M. The reliability of a modified 505 test and change-of-direction deficit time in elite youth football players. Sci. Med. Footb. 2019, 3, 157–162. ^4^

 Dugdale JH, Sanders D, Hunter AM. Reliability of Change of Direction and Agility Assessments in Youth Soccer Players. Sports (Basel). 2020 Apr 18; 8 (4): 51. doi: 10.3390/sports8040051. ^5^

 James H. Dugdale, Calum A. Arthur, Dajo Sanders & Angus M. Hunter (2019) Reliability and validity of field-based fitness tests in youth soccer players, European Journal of Sport Science, 19:6, 745-756, DOI: 10.1080/17461391.2018.1556739 ^6^

**Methods**

 The methods report the appropriate details relating to the study design, protocols, participants, and analyses performed. However, further clarity is required in a few places throughout this section (specific comments below). Further, there are a number of oversights throughout this section that require careful attention. None of these are major points, and should take little time to amend. Yet, need to be addressed prior to potential resubmissions of this manuscript.

 Figure 1: I am unsure how much this figure aids the interpretation of the study design. I personally do not feel the visual nature of this schematic clarifies the description of the trials from the text, as the study is relatively simplistic. I do not insist that the authors remove this figure, however, please consider my suggestion that it may not add value to the manuscript and may just be a “figure for the sake of a figure”. Secondly, between the trials, the authors have stated that washout periods of > 7 days were implemented. The authors should consider including the range of days (e.g. 7-14 days), as this would provide further information to the reader and allow for more accurate interpretation of the results.

 Can a citation or elaboration be provided to rationalise the >15 min rest interval between attempts?

 The term ‘respectively’ needs to be included after the explanation of the 1-10 muscle rating scale. Also, a brief sentence as to why this was collected should accompany this methodological point.

 The authors state that the warm up was the same prior to each session. I would encourage using the term ‘standardised’ when describing this in their methods to add certainty around their controls. Also, a citation needs to accompany the statement that the CMJ test was implemented to assess recovery. If this is what was intended by the insertion of citation 12, I would encourage moving this citation to the end of the sentence.

 Participant details – participant height needs to be reported to 2dp. Can the authors further elaborate on what the “School of Athletic Excellence” is? For example, what standard is this, is it affiliated to a specific school, or region performance programme? This may not be clear to readers.

 Multistage shuttle test – a brief sentence stating how performance or a score was derived is necessary (i.e. maximum number of shuttles completed, level, etc.). This information is currently not evident.

 CMJ – please state the unit of measurement for this test, and to what degree (e.g. recorded in cm to the nearest 0.1cm).

 30-m sprint and agility protocols – please separate each test into a separate sentence. At present, they appear too cramped together and it is difficult to read (particularly with reference to figures and citations within). Also, please provide citations for the original studies for the T-drill and Illinois agility tests. These need to be credited within the manuscript.

 Repeated sprint and agility protocols – were these tests performed one after the other (what I interpret from the term ‘cycles’) or were they performed independently? Clarity is required to ensure the reader understands which of these interpretations is correct.

 The sentence starting “Immediately after each repeated agility cycle…” does not make sense. Please rephrase this.

 Statistical analysis – Reference 16 seems a weak reference for this point. I encourage the authors to consider a more suitable citation for selecting this acceptable correlation cut off point.

**Results**

 The section of the results dedicated to the muscle soreness and CMJ differences (or lack thereof) should be condensed to a singular sentence and placed at the foot of the results section. This is a methodological control and not a key finding from your study, therefore, it should not feature so early and heavily within your results section. Stating that there were no differences, providing ES, and significance values would suffice.

 Table 3 – I am interested in your rationale for not providing ES to demonstrate differences (or lack thereof) for your test-retest data? This would add value in my opinion. I would also encourage the authors to present CV% data to 1dp both within this table and throughout the manuscript.

 I notice that the authors use terminology such as “excellent” or “moderate” test-retest reliability, yet they have not included these threshold within the statistical analysis section of the methods. Please include this detail so that the reader understands your criteria.

 I also note that the authors say “most RIA performance measures exhibited excellent test-retest reliability…”. Could the authors rephrase this to say “all RIA performance measures except… measures exhibited excellent test-retest reliability”? This would be more accurate and less ambiguous for the reader. This comment also applies later in this section where the authors refer to “a few variables…”.

**Discussion**

 I found the discussion well-written and thought the authors accurately interpreted and attempted to explain their findings. However, similar to the introduction, I felt this section lacked depth when discussing the main findings of the study. I encourage the authors to discuss their findings in greater depth, utilising some of the reading attached to this review.

 I encourage the authors to utilise the first paragraph of the discussion to summarise all of their main findings, and then progress to discussing each point in turn.

 The third paragraph begins “another novelty of this study”, yet this appears to be the first time novelty is addressed in the discussion?

 The fourth paragraph can draw upon a wealth of correlational studies to further support the findings observed here.

 Young WB, Dawson B, Henry GJ. Agility and Change-of-Direction Speed are Independent Skills: Implications for Training for Agility in Invasion Sports. *International Journal of Sports Science & Coaching*. 2015;10(1):159-169. doi: 10.1260/1747-9541.10.1.159 ^1^

 Spiteri T, Nimphius S, Hart NH, Specos C, Sheppard JM, Newton RU. Contribution of strength characteristics to change of direction and agility performance in female basketball athletes. J Strength Cond Res. 2014 Sep;28(9):2415-23. doi: 10.1519/JSC.0000000000000547. PMID: 24875426. ^7^

 Gabbett, T. J., Kelly, J. N., & Sheppard, J. M. (2008). Speed, Change of Direction Speed, and Reactive Agility of Rugby League Players. The Journal of Strength & Conditioning Research, 22, 174-181. http://dx.doi.org/10.1519/JSC.0b013e31815ef700 ^8^

 Sheppard, J.M.; Young, W.B.; Doyle, T.L.A.; Sheppard, T.A.; Newton, R.U. An evaluation of a new test of reactive agility and its relationship to sprint speed and change of direction speed. J. Sci Med. Sport 2006, 9, 342–349. ^9^

 The conclusion should be reframed around the key objectives of the study (i.e. that it is reliable and valid). The mention of aerobic and anaerobic energy systems seems misplaced here.

**References**

 While a variety of key texts are identified and listed within the bibliography and cited throughout the manuscript, a number of key texts are omitted. I encourage the authors to further familiarise themselves with relevant studies assessing reliability and validity of COD and agility in team sport athletes, as well as the texts identified earlier within this review.

 Dos’Santos, T.; Thomas, C.; Jones, P.A.; Comfort, P. Mechanical Determinants of Faster Change of Direction Speed Performance in Male Athletes. J. Strength Cond. Res. 2017, 31, 696–705. ^10^

 Gabbett, T.J.; Kelly, J.N.; Sheppard, J.M. Speed, change of direction speed, and reactive agility of rugby league players. J. Strength Cond. Res. 2008, 22, 174–181. ^8^

 Oliver, J.L.; Meyers, R.W. Reliability and generality of measures of acceleration, planned agility, and reactive agility. Int. J. Sports Physiol. Perform. 2009, 4, 345–354. ^11^

Is the work clearly and accurately presented and does it cite the current literature?

Partly

If applicable, is the statistical analysis and its interpretation appropriate?

Yes

Are all the source data underlying the results available to ensure full reproducibility?

Yes

Is the study design appropriate and is the work technically sound?

Yes

Are the conclusions drawn adequately supported by the results?

Yes

Are sufficient details of methods and analysis provided to allow replication by others?

Yes

Reviewer Expertise:

Fitness testing; youth athletes; talent identification; talent development; physical performance; strength and conditioning

I confirm that I have read this submission and believe that I have an appropriate level of expertise to confirm that it is of an acceptable scientific standard, however I have significant reservations, as outlined above.

## References

[1] : Agility and Change-of-Direction Speed are Independent Skills: Implications for Training for Agility in Invasion Sports. *International Journal of Sports Science & Coaching* .2015;10(1) : 10.1260/1747-9541.10.1.159 159-169 10.1260/1747-9541.10.1.159

[2] : Change of Direction and Agility Tests. *Strength and Conditioning Journal* .2018;40(1) : 10.1519/SSC.0000000000000309 26-38 10.1519/SSC.0000000000000309

[3] : Agility literature review: classifications, training and testing. *J Sports Sci* .2006;24(9) : 10.1080/02640410500457109 919-32 10.1080/02640410500457109 16882626

[4] : The reliability of a modified 505 test and change-of-direction deficit time in elite youth football players. *Science and Medicine in Football* .2019;3(2) : 10.1080/24733938.2018.1526402 157-162 10.1080/24733938.2018.1526402

[5] : Reliability of Change of Direction and Agility Assessments in Youth Soccer Players. *Sports (Basel)* .2020;8(4) : 10.3390/sports8040051 10.3390/sports8040051 32325738PMC7240391

[6] : Reliability and validity of field-based fitness tests in youth soccer players. *Eur J Sport Sci* .2019;19(6) : 10.1080/17461391.2018.1556739 745-756 10.1080/17461391.2018.1556739 30589378

[7] : Contribution of strength characteristics to change of direction and agility performance in female basketball athletes. *J Strength Cond Res* .2014;28(9) : 10.1519/JSC.0000000000000547 2415-23 10.1519/JSC.0000000000000547 24875426

[8] : Speed, change of direction speed, and reactive agility of rugby league players. *J Strength Cond Res* .2008;22(1) : 10.1519/JSC.0b013e31815ef700 174-81 10.1519/JSC.0b013e31815ef700 18296972

[9] : An evaluation of a new test of reactive agility and its relationship to sprint speed and change of direction speed. *J Sci Med Sport* .2006;9(4) : 10.1016/j.jsams.2006.05.019 342-9 10.1016/j.jsams.2006.05.019 16844413

[10] : Mechanical Determinants of Faster Change of Direction Speed Performance in Male Athletes. *J Strength Cond Res* .2017;31(3) : 10.1519/JSC.0000000000001535 696-705 10.1519/JSC.0000000000001535 27379954

[11] : Reliability and Generality of Measures of Acceleration, Planned Agility, and Reactive Agility. *International Journal of Sports Physiology and Performance* .2009;4(3) : 10.1123/ijspp.4.3.345 345-354 10.1123/ijspp.4.3.345 19953822

# Response

**Published:** 2021-09-27

**Authors:** Kenji Doma

**Affiliations:** James Cook University, Australia

Reviewer’s comment: The present study aimed to explore the validity and reliability of repeated “agility” tests in a sample of youth rugby players. The study provides interesting data for an applied audience and is methodologically sound and accurate in the presentation of findings. This being said, I have identified a range of areas that should be improved prior to potential resubmissions of this manuscript. For example, the introduction (and study in its entirety) is inaccurate in its use of the term “agility” (opposed to COD which the authors actually assess). Furthermore, the introduction needs to be further developed to support the scope of the study. The authors need to create more of a rationale for running such a large number of correlations – exploring correlational analyses needs to be warranted based upon an assumption of a relationship (or lack thereof) between variables. In its current form, I am unsure whether this manuscript provides such rationale. The methods and results are accurate, but with a large number of small errors. Finally, the discussion addresses key discussion points formulated from the findings of the study, but similar to the introduction, lacks some critical depth. I do think that the authors have a publishable study here, however, a number of revisions will be required before I would be happy to approve this manuscript for indexing. I hope that my comments are received as I intend them and are useful in further strengthening this manuscript. I hope to receive the opportunity to review this manuscript again in its revised form.

**Author’s response: Dear reviewer, thank you for your comments on the paper. We agree that your comments have improved the quality of our work.**

 Abstract

 Reviewer’s comment: The abstract is concise and accurate to the details of the study. I have just a couple of minor comments (below):

 The initial sentence is accurate and outlines the requirement of the study. However, I would encourage the authors to insert a word such as ‘simultaneously’ or ‘congruently’, or attempt to capture the lack of a suitable test to capture the performance of these abilities in combination.

**Author’s response: The word ‘simultaneously’ has been included.**

 Reviewer’s comment: Abstract Methods – the inclusion of the repeated T-agility test appears suddenly with no prior reference. If this is a key concentration of your study, I would encourage to insert earlier in the abstract along with the RIA.

**Author’s response: Repeated T-agility protocol now mentioned in the Background.**

 Reviewer’s comment: Abstract Results – please specify exact p values rather than just <0.05. CV% can be presented to 1dp for brevity.

**Author’s response: We were unable to provide exact p-values, as several outcome measures from the repeated COD protocols, such as average time, best time, total time etc were presented as “performances”. This same approach was used for the r-values as well to ensure the word limit did not exceed journal guidelines. However, we did present the CV% to 1dp as requested.**

 Introduction

 Reviewer’s comment: The introduction is short and concise. However, I feel some key information that would further support the rationale for your study has been omitted. I would encourage the authors to further develop this section. A suggestion would be to expand the second paragraph into two: the first discussing the validity aspects, the second the reliability aspects. I do not expect these changes to be too onerous, but believe it would strengthen this section of your manuscript if you were to implement these changes. I have some further specific comments below.

**Author’s response: The second paragraph has been further developed into two as requested, with the first focusing on reliability and the second on validity.**

 Reviewer’s comment: The opening sentence would read better if it were split into two separate sentences.

**Author’s response: We have simplified the first sentence by removing the context of ‘agility’, as we have converted all ‘agility’ terms to ‘change-of-direction’.**

 Reviewer’s comment: The authors should attempt to clarify during opening paragraph that they are discussing change of direction (COD) performance, rather than agility. Although COD performance is a key component of agility performance, the lack of an external stimuli prevents classification of agility. Although the tests identified by the authors in this opening paragraph have titled themselves ‘agility’ tests, by definition they are not. The authors should be careful in their stance here as the literature has evolved substantially since the inception of tests such as these, and appropriate classification and terminology is essential for the present study.

 Young, W.; Dawson, B.; Henry, G. Agility and Change-of-Direction Speed are Independent Skills: Implications for Agility in Invasion Sports. Int. J. Sports Sci. Coach. 2015, 10, 159–169.1

 Nimphius, S.; Callaghan, S.J.; Bezodis, N.E.; Lockie, R.G. Change of Direction and Agility Tests: Challenging Our Current Measures of Performance. Strength Cond. J. 2018, 40, 26–38. 2

 Sheppard, J.M.; Young, W.B. Agility literature review: Classifications, training and testing. J. Sports Sci. 2006, 24, 919–932.3

**Author’s response: Thank you for your suggestion. As mentioned earlier, we have changed all ‘agility’ terms into ‘change-of-direction’.**

 Reviewer’s comment: Regarding the reliability aspect of the study, two recent papers have suggested that reliability of COD and agility may be lower in adolescent and youth athletes (as per your sample). Although these authors observed this during maximal COD/agility performance, opposed to repeated as in the present study, I believe this would be worth including and would further warrant the exploration of your research question and study.

 Taylor, J.M.; Cunningham, L.; Hood, P.; Thorne, B.; Irvin, G.; Weston, M. The reliability of a modified 505 test and change-of-direction deficit time in elite youth football players. Sci. Med. Footb. 2019, 3, 157–162.4

 Dugdale JH, Sanders D, Hunter AM. Reliability of Change of Direction and Agility Assessments in Youth Soccer Players. Sports (Basel). 2020 Apr 18; 8 (4): 51. doi: 10.3390/sports8040051.5

 James H. Dugdale, Calum A. Arthur, Dajo Sanders & Angus M. Hunter (2019) Reliability and validity of field-based fitness tests in youth soccer players, European Journal of Sport Science, 19:6, 745-756, DOI: 10.1080/17461391.2018.15567396

**Author’s response: Thank you for your suggestion, we have included the references above in the paragraph explaining studies on the reliability of COD protocols.**

 Methods

 Reviewer’s comment: The methods report the appropriate details relating to the study design, protocols, participants, and analyses performed. However, further clarity is required in a few places throughout this section (specific comments below). Further, there are a number of oversights throughout this section that require careful attention. None of these are major points, and should take little time to amend. Yet, need to be addressed prior to potential resubmissions of this manuscript.

**Author’s response: Thank you for your comments, we have addressed these below.**

 Reviewer’s comment: Figure 1: I am unsure how much this figure aids the interpretation of the study design. I personally do not feel the visual nature of this schematic clarifies the description of the trials from the text, as the study is relatively simplistic. I do not insist that the authors remove this figure, however, please consider my suggestion that it may not add value to the manuscript and may just be a “figure for the sake of a figure”. Secondly, between the trials, the authors have stated that washout periods of > 7 days were implemented. The authors should consider including the range of days (e.g. 7-14 days), as this would provide further information to the reader and allow for more accurate interpretation of the results.

**Author’s response: Thank you for your comment. We have decided to keep the figure to ensure clarity as we used a cross-over randomized design, which is unusual for a study examining the reliability and validity of protocols. We have included 7-14 days as a washout period as suggested in the figure.**

 Reviewer’s comment: Can a citation or elaboration be provided to rationalise the >15 min rest interval between attempts?

**Author’s response: A citation has been included with a study that used a 15-minute recovery period in-between repeated sprint and COD protocols in the one testing session.**

 Reviewer’s comment: The term ‘respectively’ needs to be included after the explanation of the 1-10 muscle rating scale. Also, a brief sentence as to why this was collected should accompany this methodological point.

**Author’s response: Included as requested.**

 Reviewer’s comment: The authors state that the warm up was the same prior to each session. I would encourage using the term ‘standardised’ when describing this in their methods to add certainty around their controls. Also, a citation needs to accompany the statement that the CMJ test was implemented to assess recovery. If this is what was intended by the insertion of citation 12, I would encourage moving this citation to the end of the sentence.

**Author’s response: The term ‘standardised’ has been included when describing the warm-up as requested in the Methods section. The citation explaining the use of CMJ to determine recovery dynamics was moved to the end of the sentence as requested.**

 Reviewer’s comment: Participant details – participant height needs to be reported to 2dp. Can the authors further elaborate on what the “School of Athletic Excellence” is? For example, what standard is this, is it affiliated to a specific school, or region performance programme? This may not be clear to readers.

**Author’s response: The participant height is reported to 2dp (1.77m). Further information on the excellence program has been included.**

 Reviewer’s comment: Multistage shuttle test – a brief sentence stating how performance or a score was derived is necessary (i.e. maximum number of shuttles completed, level, etc.). This information is currently not evident.

**Author’s response: The VO2max was estimated based on the level completed, which has now been included in the Methods section.**

 Reviewer’s comment: CMJ – please state the unit of measurement for this test, and to what degree (e.g. recorded in cm to the nearest 0.1cm).

**Author’s response: The CMJ was recorded to the full cm, not to the nearest 0.1cm. This has now been clarified.**

 Reviewer’s comment: 30-m sprint and agility protocols – please separate each test into a separate sentence. At present, they appear too cramped together and it is difficult to read (particularly with reference to figures and citations within). Also, please provide citations for the original studies for the T-drill and Illinois agility tests. These need to be credited within the manuscript.

**Author’s response: The sentences for the description of the protocols have been separated as requested. The original studies have also been included as requested.**

 Reviewer’s comment: Repeated sprint and agility protocols – were these tests performed one after the other (what I interpret from the term ‘cycles’) or were they performed independently? Clarity is required to ensure the reader understands which of these interpretations is correct.

**Author’s response: We have reworded the term ‘cycles’ to ‘repetitions’ to improve clarity. As mentioned in Research Design and in Figure 1, RSA was conducted in Session 2, whilst RTT and RIA were conducted in the same session with 15-min rest in-between each protocol. The Figure reinforces this design, and rewording ‘cycles’ to ‘repetitions’ improves the clarity of the protocols.**

 Reviewer’s comment: The sentence starting “Immediately after each repeated agility cycle…” does not make sense. Please rephrase this.

**Author’s response: This has been reworded to, “The participant’s heart rate (HR, Polar Heart Rate Monitor, Polar H10, Finland) and rating of perceived-exertion (RPE, Borg category scale 1-10) were collected at the completion of each repetition of the RSA, RTT and RIA protocols. The maximum and average HR and RPE values were then reported from the 6 repetitions”**

 Reviewer’s comment: Statistical analysis – Reference 16 seems a weak reference for this point. I encourage the authors to consider a more suitable citation for selecting this acceptable correlation cut off point.

**Author’s response: Additional reference included as requested.**

 Results

 Reviewer’s comment: The section of the results dedicated to the muscle soreness and CMJ differences (or lack thereof) should be condensed to a singular sentence and placed at the foot of the results section. This is a methodological control and not a key finding from your study, therefore, it should not feature so early and heavily within your results section. Stating that there were no differences, providing ES, and significance values would suffice.

**Author’s response: Simplified and included at the end of the Results section as requested.**

 Reviewer’s comment: Table 3 – I am interested in your rationale for not providing ES to demonstrate differences (or lack thereof) for your test-retest data? This would add value in my opinion. I would also encourage the authors to present CV% data to 1dp both within this table and throughout the manuscript.

**Author’s response: Thank you for your comment. The ES was not reported as we based the measurement error on CV, with larger CV% values exhibiting greater measurement error and poorer test-retest reliability. In our opinion, inclusion of ES would not provide any further clarity about the reliability of the tests, beyond that of CV.**

 Reviewer’s comment: I notice that the authors use terminology such as “excellent” or “moderate” test-retest reliability, yet they have not included these threshold within the statistical analysis section of the methods. Please include this detail so that the reader understands your criteria.

**Author’s response: Included as requested.**

 Reviewer’s comment: I also note that the authors say “most RIA performance measures exhibited excellent test-retest reliability…”. Could the authors rephrase this to say “all RIA performance measures except… measures exhibited excellent test-retest reliability”? This would be more accurate and less ambiguous for the reader. This comment also applies later in this section where the authors refer to “a few variables…”.

**Author’s response: Thank you for your suggestion. We have reworded this to, “Most RIA measures exhibited excellent test-retest reliability (ICC = 0.92-0.97), good levels of agreement (ratio LOA = 1.05-1.06) and low measurement error (CV = 2.17 - 2.68%) (Table 3). However, FI and average RPE demonstrated moderate test-retest reliability (ICC = 0.87 and 0.76, respectively), poorer levels of agreement (ratio LOA = 2.57 and 2.23, respectively) and higher measurement error (CV = 25.3 and 15.8%, respectively, Table 3).” The next paragraph has been rewritten to, “For the RTT, excellent test-retest reliability (ICC = 0.91), good levels of agreement (ratio LOA = 1.08) and low measurement error (CV = 2.17-2.68%) were identified for TT, BT and AT (Table 3), although maximum RPE demonstrated higher levels of measurement error (CV = 12.3%) between Sessions 4 and 5 (Table 3).”**

 Discussion

 Reviewer’s comment: I found the discussion well-written and thought the authors accurately interpreted and attempted to explain their findings. However, similar to the introduction, I felt this section lacked depth when discussing the main findings of the study. I encourage the authors to discuss their findings in greater depth, utilising some of the reading attached to this review.

**Author’s response: Thank you for your comments. The Discussion has been adjusted to address the comments below.**

 Reviewer’s comment: I encourage the authors to utilise the first paragraph of the discussion to summarise all of their main findings, and then progress to discussing each point in turn.

**Author’s response: Summary of findings included as requested in the first paragraph.**

 Reviewer’s comment: The third paragraph begins “another novelty of this study”, yet this appears to be the first time novelty is addressed in the discussion?

**Author’s response: This specific text has been deleted to improve clarity.**

 Reviewer’s comment: The fourth paragraph can draw upon a wealth of correlational studies to further support the findings observed here.

 Young WB, Dawson B, Henry GJ. Agility and Change-of-Direction Speed are Independent Skills: Implications for Training for Agility in Invasion Sports. International Journal of Sports Science & Coaching. 2015;10(1):159-169. doi:10.1260/1747-9541.10.1.1591

 Spiteri T, Nimphius S, Hart NH, Specos C, Sheppard JM, Newton RU. Contribution of strength characteristics to change of direction and agility performance in female basketball athletes. J Strength Cond Res. 2014 Sep;28(9):2415-23. doi: 10.1519/JSC.0000000000000547. PMID: 24875426.7

 Gabbett, T. J., Kelly, J. N., & Sheppard, J. M. (2008). Speed, Change of Direction Speed, and Reactive Agility of Rugby League Players. The Journal of Strength & Conditioning Research, 22, 174-181. http://dx.doi.org/10.1519/JSC.0b013e31815ef7008

 Sheppard, J.M.; Young, W.B.; Doyle, T.L.A.; Sheppard, T.A.; Newton, R.U. An evaluation of a new test of reactive agility and its relationship to sprint speed and change of direction speed. J. Sci Med. Sport 2006, 9, 342–349.9

**Author’s response: A sentence with relevant references listed above were included as requested.**

 Reviewer’s comment: The conclusion should be reframed around the key objectives of the study (i.e. that it is reliable and valid). The mention of aerobic and anaerobic energy systems seems misplaced here.

**Author’s response: A sentence on the validity of the RIA protocols has been included.**

 References

 Reviewer’s comment: While a variety of key texts are identified and listed within the bibliography and cited throughout the manuscript, a number of key texts are omitted. I encourage the authors to further familiarise themselves with relevant studies assessing reliability and validity of COD and agility in team sport athletes, as well as the texts identified earlier within this review.

 Dos’Santos, T.; Thomas, C.; Jones, P.A.; Comfort, P. Mechanical Determinants of Faster Change of Direction Speed Performance in Male Athletes. J. Strength Cond. Res. 2017, 31, 696–705.10

 Gabbett, T.J.; Kelly, J.N.; Sheppard, J.M. Speed, change of direction speed, and reactive agility of rugby league players. J. Strength Cond. Res. 2008, 22, 174–181.8

 Oliver, J.L.; Meyers, R.W. Reliability and generality of measures of acceleration, planned agility, and reactive agility. Int. J. Sports Physiol. Perform. 2009, 4, 345–354.11

**Author’s response: Thank you for kindly providing the references. A number of these have been included in the paper as requested.**
